# K‐CC‐MoCo: A Fast *k*‐Space‐Based Respiratory Motion Correction for Highly Accelerated First‐Pass Perfusion Cardiovascular MR

**DOI:** 10.1002/mrm.70287

**Published:** 2026-02-09

**Authors:** Elisa Moya‐Sáez, Rosa‐María Menchón‐Lara, Javier Sánchez‐González, Catarina N. Carvalho, Andreia S. Gaspar, Carlos Real, Carlos Galán‐Arriola, Rita G. Nunes, Borja Ibanez, Teresa M. Correia, Carlos Alberola‐López

**Affiliations:** ^1^ ETSI de Telecomunicación, Universidad de Valladolid Valladolid Spain; ^2^ Instituto de Investigación Biosanitaria de Valladolid (IBioVALL) Valladolid Spain; ^3^ Universidad Politécnica de Cartagena Member of European University of Technology EUT+ Cartagena Spain; ^4^ Philips Healthcare Iberia Madrid Spain; ^5^ Center of Marine Sciences‐CCMAR Faro Portugal; ^6^ Institute for Systems and Robotics ‐ Lisboa and Department of Bioengineering Instituto Superior Técnico, Universidade de Lisboa Lisbon Portugal; ^7^ Spanish National Centre for Cardiovascular Research Madrid Spain; ^8^ Department of Cardiology Hospital Universitario Clínico San Carlos Madrid Spain; ^9^ School of Biomedical Engineering and Imaging Sciences King's College London London UK

**Keywords:** motion correction, myocardial first‐pass perfusion, respiratory motion, rigid registration

## Abstract

**Purpose:**

First‐pass perfusion cardiovascular MR (FPP‐CMR) enables the non‐invasive diagnosis of microcirculation and coronary artery disease. In free‐breathing FPP‐CMR, motion correction is usually performed in the image domain, requiring an initial reconstruction. This fact hinders its use in model‐based and deep learning reconstructions, which present remarkable performance in obtaining high‐quality images from highly accelerated acquisitions. We address this challenge by estimating and correcting respiratory motion in free‐breathing FPP‐CMR directly in *k*‐space.

**Methods:**

We propose K‐CC‐MoCo, an inter‐frame rigid motion correction approach formulated exclusively in *k*‐space that handles dynamic contrast through a specifically targeted design of the normalized cross‐correlation (CC) objective function to deal with the dynamic contrast. In addition, an ROI‐based coil‐compression approach was employed to focus the optimization on the heart region. The proposed method was compared to state‐of‐the‐art image‐based registration using a digital phantom and real free‐breathing acquisitions with different accelerations.

**Results:**

The proposed *k*‐space‐based method is approximately 2× faster and can correct respiratory motion even at high acceleration factors (up to 50×), where the image‐based method fails due to severe undersampling artifacts. Notably, after K‐CC‐MoCo, the time‐averaged images are visibly less blurred. Quantitative metrics (SSIM, etc.) support this conclusion.

**Conclusion:**

K‐CC‐MoCo outperforms image‐based correction in free‐breathing FPP‐CMR acquisitions accelerated up to 50×. Respiratory motion is estimated and corrected in *k*‐space, enabling its use for model‐based and/or deep learning reconstructions from highly accelerated scans.

## 1 Introduction

First‐pass perfusion cardiovascular MR (FPP‐CMR) enables non‐invasive detection of myocardial perfusion abnormalities caused by both epicardial coronary stenosis and microvascular dysfunction [1, 2]. An FPP‐CMR protocol involves acquiring a sequence of contrast‐enhanced images during the rapid passage of a contrast bolus through the heart [3]. The assessment of dynamic images is routinely performed visually by skilled reporters; however, quantitative FPP‐CMR has emerged as a more objective approach for detecting perfusion defects by converting MR signal intensity changes into maps of myocardial blood flow.

In a conventional FPP‐CMR protocol, there is a trade‐off between heart coverage, and both temporal and spatial resolution; typically, 3–4 short‐axis slices are acquired with approximately 2.5 mm^2^ in‐plane spatial resolution in a total time of approximately 60 s [3]. To deal with cardiac motion, data acquisition is commonly synchronized with the electrocardiogram (ECG) signal to ensure that images for each slice are captured during the same cardiac position, and patients are instructed to hold their breath to minimize frame misalignments caused by respiratory motion. Recently, there is a growing preference for acquiring images during free‐breathing, as it offers increased reliability, particularly for ill patients who struggle to hold their breath, and enhanced patient comfort [4, 5]. Consequently, the integration of respiratory motion correction (MoCo) approaches before the myocardial perfusion quantification is essential in free‐breathing acquisitions. Also, for addressing residual motion resulting from suboptimal breath‐holding, particularly during adenosine‐induced stress protocols.

Additionally, to improve resolution and coverage, data should be acquired as fast as possible. One of the main acceleration strategies consists of undersampling the *k*‐space over time below the Nyquist limit followed by a specific reconstruction method such as *k*‐*t* SENSE [6], compressed sensing (CS) [7] or low‐rank plus sparse (L + S) [8] among others. Recently, deep learning and model‐based reconstruction methods have taken the stage, demonstrating unprecedented results in producing high‐quality images from highly accelerated acquisitions [9, 10, 11]. However, in the presence of respiratory motion, images produced by these techniques can display considerable degradation in image quality, once again highlighting the importance of MoCo.

Motion can be accounted for either prospectively or retrospectively. Prospective respiratory MoCo has been performed using prospective slice tracking (PST) [12]; however, this technique requires a dedicated navigator setup and is susceptible to the quality of the navigator signal. Additionally, the precision of the PST correction is affected in stress protocols due to the higher heart rate and changes in respiratory motion [13]. FastNAV [14] is also a prospective MoCo method based on a right hemidiaphragm (RHD) navigator with a subject‐specific tracking factor instead of a fixed one. However, it requires a calibration process that has only been tested on rest protocols, not in stress [14].

Regarding retrospective MoCo, various methods have been developed for FPP‐CMR, which use multimodal metrics to deal with image intensity changes caused by the dynamic contrast. Typically, motion estimation occurs in the image domain using image‐based registration algorithms. Thus, the obtained deformation fields can be modeled as either rigid [15, 16] or non‐rigid [17, 18, 19, 20, 21] transformations. Non‐rigid registration methods should theoretically provide better correction [22]; however, they may be more sensitive to variations in signal‐to‐noise ratio (SNR), contrast‐to‐noise ratio (CNR), and are prone to blurring and geometric distortions.

The common pipeline for obtaining motion‐corrected images consists of an initial reconstruction to obtain dynamic images that enable the estimation of motion followed by a subsequent motion‐compensated reconstruction [4, 5, 23, 24, 25, 26] In some cases, this process is performed iteratively several times [27]. This pipeline is not only time‐consuming but also difficult to integrate with model‐based and deep learning reconstruction methods.

Alternatively, motion could be retrospectively estimated/corrected directly in the *k*‐space domain, avoiding the need for an initial reconstruction, which notably reduces the computational burden. Additionally, MoCo methods in *k*‐space could potentially achieve increased performance at high acceleration factors (AFs), where initially‐reconstructed images present poor quality. Although some methods for *k*‐space‐based MoCo have been proposed, these have not been applied to undersampled FPP‐CMR data [28, 29, 30, 31]. Specifically, Huttinga et al. [28] proposed a forward model in which the *k*‐space value at a given time instant is generated from the warped version of a reference object whose image is available. However, the requirement of a prior available reference image may represent a limitation of this approach. More recently, Olausson et al. [32] proposed an extension of the MR‐MOTUS framework to cardiac MR, in which the reference image is not assumed to be static, thereby accommodating contrast changes, for example. In this method, the solution iteratively alternates between estimating the deformation field and reconstructing the motion‐corrected images.

In this work, we propose K‐CC‐MoCo, a cross‐correlation (CC) based rigid inter‐frame respiratory MoCo method for highly accelerated FPP‐CMR data formulated exclusively in *k*‐space. Specifically, K‐CC‐MoCo aims to eliminate the effect of respiration motion in free‐breathing ECG‐triggered FPP‐CMR by aligning the heart position along the different frames. The method was tested on a digital phantom, a DICOM dataset with 40 patients, and also on raw *k*‐space data. The experiments were performed with AFs from 10× to 50×.

## 2 Theory

Let $I_{f}\left(r\right)$ and $I_{c}\left(r\right)$, denote two complex images. The cross‐correlation (CC) between them can be expressed as

(1) $$\mathrm{CC}\left(I_{f},I_{c}\right)\left(r\right)=I_{f}\left(r\right)⋆I_{c}^{*}\left(-r\right),$$

where $r\in {\mathbb{Z}}^{2}$ denotes the spatial coordinate, ⋆ is used for convolution and 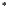 is the complex conjugate. Thus, according to the convolution theorem, CC can also be computed in the Fourier domain

(2) $$ℱ\left\{\mathrm{CC}\left(I_{f},I_{c}\right)\left(r\right)\right\}=S_{f}\left(k\right)S_{c}^{*}\left(k\right),$$

where $S_{f}\left(k\right)$ and $S_{c}\left(k\right)$ denote the *k*‐space versions of images $I_{f}\left(r\right)$ and $I_{c}\left(r\right)$, respectively, and $k\in {\mathbb{Z}}^{2}$ denotes the *k*‐space coordinate.

If we take the inverse Fourier transform, we obtain

(3) $${ℱ}^{-1}\left\{ℱ\left\{\mathrm{CC}\left(I_{f},I_{c}\right)\left(r\right)\right\}\right\}=\frac{1}{N}\sum_{k}S_{f}\left(k\right)S_{c}^{*}\left(k\right)e^{j2\pi \mathrm{kr}},$$

where N represents the total number of samples. Since this equation is valid for all values of r, we can set r=0, which yields

(4) $$\mathrm{CC}\left(I_{f},I_{c}\right)=\mathrm{CC}\left(S_{f},S_{c}\right)=\frac{1}{N}\sum_{k}S_{f}\left(k\right)S_{c}^{*}\left(k\right)$$

Correspondingly, we can also define the normalized cross‐correlation (NCC) in *k*‐space as

(5) $$\mathrm{NCC}\left(S_{f},S_{c}\right)=\frac{\sum_{k}S_{f}\left(k\right)S_{c}^{*}\left(k\right)}{\sqrt{\sum_{k}{\left|S_{f}\left(k\right)\right|}^{2}\sum_{k}{\left|S_{c}\left(k\right)\right|}^{2}}},$$

where the denominator is expressed in *k*‐space according to Parseval's theorem.

In this work, to be robust against the strong intensity variations caused by the dynamic contrast, we define a Gaussian‐normalized cross‐correlation (GNCC) in *k*‐space computed between the reference *k*‐space $S_{f}\left(k\right)$ and the corrected *k*‐space $S_{c}\left(k;\Theta \right)$ corresponding to each frame. Specifically, the registration metric employed is

(6) $$\begin{array}{ll} & \text{GNCC}\left(S_{f},S_{c}\right)\overset{\Delta }{=}\\ & \frac{\sum_{k}\left(S_{f}\left(k\right)-S_{f}\left(k\right)G\left(k\right)\right)\left(S_{c}^{*}\left(k;\Theta \right)-S_{c}^{*}\left(k;\Theta \right)G\left(k\right)\right)}{\sqrt{\sum_{k}{\left|S_{f}\left(k\right)-S_{f}\left(k\right)G\left(k\right)\right|}^{2}\sum_{k}{\left|S_{c}(k;\Theta )-S_{c}(k;\Theta )G\left(k\right)\right|}^{2}}}\\ & =V\left(\Theta \right) \end{array}$$

where G(k) is a Gaussian function used to minimize the influence of contrast changes located in the center of the *k*‐space. The corrected *k*‐space $S_{c}\left(k;\Theta \right)$ is defined as the rigidly corrected version of the moving *k*‐space $S_{m}\left(k\right)$ with a rigid transformation defined by parameters $\Theta =\left[\begin{array}{c} \Theta_{R}\\ \Theta_{T} \end{array}\right]$, where subscript R stands for rotation and T for translation. For the particular case of a 2‐dimensional (2D) correction, these parameters are defined by $\Theta_{R}=\left[\theta_{1}\right]$ and $\Theta_{T}={\left[\begin{array}{cc} \theta_{2} & \theta_{3} \end{array}\right]}^{T}$.

Thus, the corrected *k*‐space is defined as

(7) $$S_{c}\left(k;\Theta \right)=S_{m}\left(R^{T}k\right)e^{j2\pi \Theta_{T}^{T}k},$$

where $R^{T}$ is the inverse of a rotation matrix with parameters $\Theta_{R}$ and $S_{m}\left(k\right)$ denotes the *k*‐space values of the original *moving* image. Note that we exploit the fact that a rotation and a translation of the object in image‐space corresponds to an identical rotation and a linear phase‐shift of its Fourier transform in *k*‐space, respectively.

Then, the minimization problem is formulated as

(8) $$\hat{\Theta }=\arg\min_{\Theta }\;-{\left|V\left(\Theta \right)\right|}^{2}$$

A detailed formulation of the approach can be found in Appendix A.

## 3 Methods

### 3.1 Data

#### 3.1.1 Digital Phantom

Fully‐sampled FPP‐CMR data was generated with MRXCAT [33] using the following parameters: one slice, FOV = 320 × 320 mm^2^, in‐plane resolution = 1.6 × 1.6 mm^2^, slice thickness = 5 mm, TR/TE/TS = 2/1/150 ms, flip angle $=15^{{}^{\circ}}$, contrast agent dose = 0.075 mmol/kg, contrast agent relaxivity = 5.6 L/mmol/s, and 32 time frames. Different noise levels were simulated on the data using MRXCAT with different values of the contrast‐to‐noise ratio (CNR) parameter*: *Inf* (i.e., without noise), 20, 10, 5, and 1. Afterwards, sensitivity maps for 16 receiver coils were simulated. One thousand instances of random translations (between −4 and 4 voxels) and rotations (between 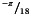 and 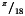 rad) are independently simulated in the *k*‐space of each frame. In addition, to model different breathing patterns, we simulated a motion scenario in which the foot–head translation parameter followed an asymmetric sawtooth pattern, while the other two parameters were set to zero. Two motion amplitudes were evaluated for the varying parameter to model shallow and deep breathing patterns. Specifically, the translation parameter $\theta_{3}$ varied between −1 and 1 voxels and between −4 and 4 voxels, respectively. *K*‐space data were undersampled following a golden‐angle radial (*k*,*t*)‐sampling in Cartesian grid [34, 35] with 32, 16, 8, and 4 spokes per frame, which corresponds to AF of approximately 6×, 10×, 20×, and 45×,^†^ respectively. For the sake of completeness, we also tried different sampling patterns; thus, *k*‐space data were also undersampled following a golden‐angle pseudo‐spiral sampling [36] with 16, 10, and 6 spirals per frame (i.e., AFs of 35×, 55× and 90×) and a Gaussian variable‐density random undersampling along the phase encoding direction with 20, 10, and 5 lines per frame (i.e., AFs of 10×, 20× and 40×).

#### 3.1.2 Undersampled DICOM Dataset

Forty patients underwent a free‐breathing REST/STRESS FPP‐CMR protocol at CNIC with Institutional Review Board (IRB) approval. Data were acquired on a Philips Elition X 3T MRI scanner (Philips Healthcare, Best, The Netherlands) using the following parameters: three slices, FOV = 300 × 300 mm^2^, in‐plane resolution = 2.6 × 2.6 mm^2^, in‐plane reconstruction resolution = 1.35 × 1.35, slice thickness = 10 mm, TR/TE/TS = 2.45/1.15/80 ms, flip angle = 15°, compressed sensing = 2.3, partial Fourier of 75%, scan time = 60 s, and approximately 60 and 90 time frames for REST and STRESS, respectively. In this work, only the mid slice was employed. In addition, only DICOM data was available; thus, sensitivity maps for 16 receiver coils were simulated. Afterwards, *k*‐space data were retrospectively generated from multi‐coil images and undersampled following a golden‐angle radial (*k*,*t*)‐sampling in Cartesian positions with AF ranging from 25× (8 spokes per frame) to 50× (4 spokes per frame)^†^.

#### 3.1.3 Undersampled Raw *k*‐Space Dataset

A free‐breathing REST FPP‐CMR acquisition was performed in one patient with IRB approval. FPP‐CMR acquisition was conducted in a Philips Elition X 3T MRI scanner (Philips Healthcare, Best, The Netherlands) with the following parameters: 64 frames, three slices, FOV = 350 × 350 mm^2^, in‐plane resolution = 2.0 × 2.0 mm^2^, slice thickness = 10 mm, TR/TE/TS = 2.57/1.27/194.06 ms, flip angle = 15°. A total of six coil‐compressed virtual channels were used from 28 independent coil elements.

Acquisition was performed following a golden‐angle radial (*k*,*t*)‐sampling scheme with 27 spokes per frame. Afterwards, different undersamplings were carried out in a radial basis, namely, with 14, 9, and 5 spokes per frame (i.e., AF of 15×, 22×, 40×, respectively) changing the sampling pattern from each dynamic scan. The k‐space was then gridded to a Cartesian grid and the coil sensitivity maps were estimated using ESPIRIT [37].

### 3.2 K‐CC‐MoCo

**FIGURE 1:**
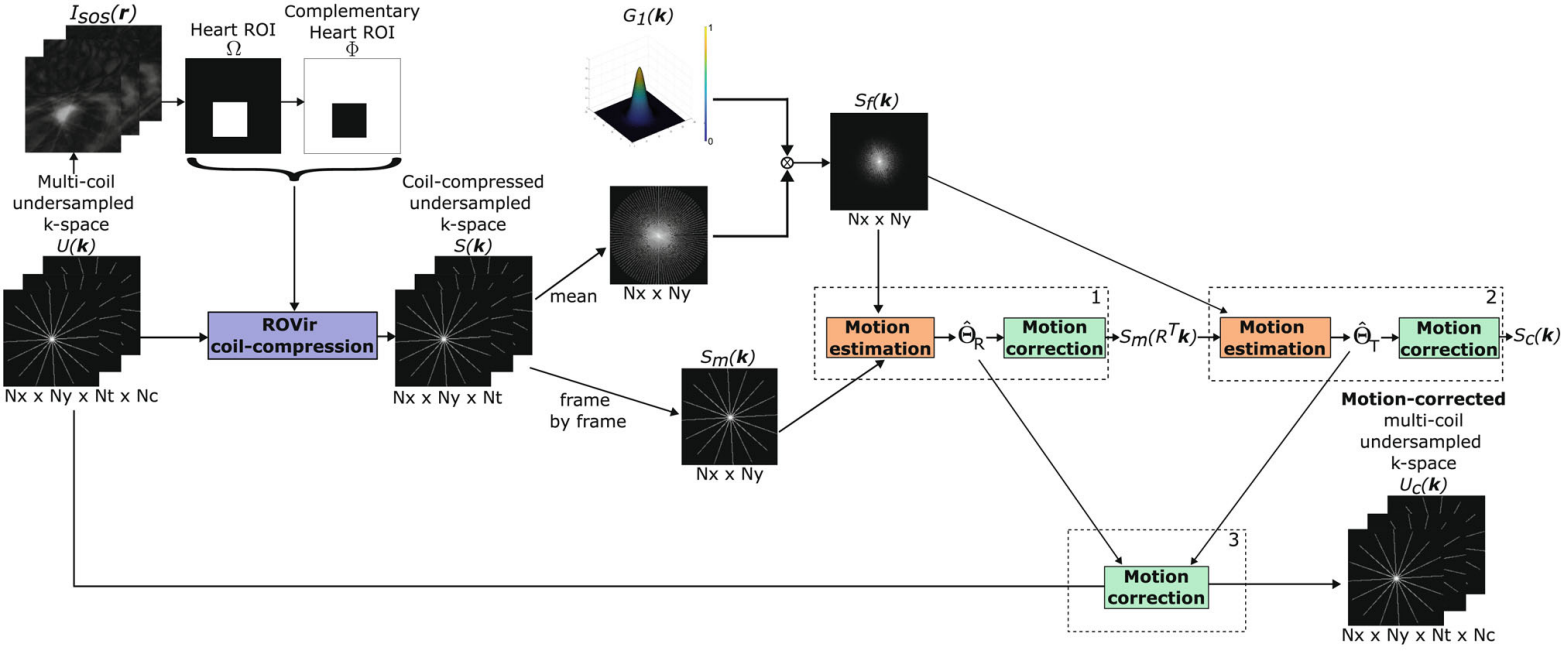
Pipeline of the proposed K‐CC‐MoCo approach. Using a sum‐of‐squares (SoS) approach, the multi‐coil undersampled *k*‐space U(k) is reconstructed into a dynamic image $I_{\mathrm{SoS}}$ in which an ROI of the heart region is detected. Next, ROVir coil‐compression is performed to generate a single virtual coil *k*‐space S(k), in which the signal from the heart ROI Ω is maximized, and the signal outside Ω (i.e., Φ ROI) is minimized. Then, the *k*‐space of each frame $S_{m}\left(k\right)$ is rigidly registered to the synthetic reference $S_{f}\left(k\right)$ (i.e., pointwise *k*‐space mean across time weighted by a Gaussian filter $G_{1}\left(k\right)$). Lastly, the multi‐coil undersampled *k*‐space is motion‐corrected $U_{c}\left(k\right)$ with $\Theta_{R}$ and $\Theta_{T}$ parameters previously estimated.

The proposed approach performs pairwise registration in the *k*‐space domain to align each frame of the dynamic images to a common reference, following the pipeline represented in Figure 1. The registration is performed against a synthetic reference computed as the pointwise *k*‐space mean across time. In this manner, we pursue the following goals: (a) to enhance the robustness of the algorithm by capturing overall contrast and position, and (b) to artificially compensate for the missing information by generating a synthetic reference with a reduced undersampling factor. In addition, this synthetic reference is weighted by a Gaussian filter $G_{1}\left(k\right)$, which was used to damp the influence of high spatial frequencies given than, due to rotating radial sampling pattern, only a few samples coincide at the same position. The standard deviation (σ) of the Gaussian $G_{1}\left(k\right)$ was empirically set to 20 Δk units.

To focus the cost function on the heart region, we used a region‐optimized virtual (ROVir) coils approach [38] to compress multi‐coil *k*‐space into a single virtual coil in which the ROI signal is maximized and the signal outside the ROI is minimized. Note that respiratory motion implies, to a large extent, a rigid transformation of the heart region, although smaller elastic deformations may also occur. However, outside the heart region, for example, in the abdominal cavity, the deformations caused by respiration are highly non‐rigid. Thus, in order to identify the heart region, we employed a simple sum‐of‐squares (SoS) approach, that is, root‐mean‐square average of the zero‐filled images associated with the different coils, to reconstruct the multi‐coil undersampled *k*‐space into a dynamic image in which the ROI of the heart is detected. This ROI can be manually delineated or automatically identified; for the latter, we computed the standard deviation from all frames and, afterwards, the largest connected region with higher standard deviation was detected. An square ROI (defined as Ω), whose size was empirically set to 80 × 80 pixels, was selected around this connected region.

ALGORITHM 1 **Pseudocode for K‐CC‐MoCo (See Figure 1).**

**Inputs:**

U(k): multi‐coil undersampled *k*‐space

**Steps:**

Reconstruct the sum of squares (SoS) image $I_{\mathrm{SoS}}\left(r\right)$ from U(k).

Compute the ROI of the heart Ω on the $I_{\mathrm{SoS}}\left(r\right)$

Compute the ROI Φ complementary to Ω

Compress U(k) into a single virtual coil *k*‐space S(k) using the ROVir approach [38] with ROIs Ω and Φ

Compute the Gaussian function $G_{1}\left(k\right)$ with σ=20

Compute the synthetic reference *k*‐space $S_{f}\left(k\right)$ as the *k*‐space mean over time of S(k) weighted by $G_{1}\left(k\right)$

**for** each frame **do**

  Select the *k*‐space of the current frame $S_{m}\left(k\right)$ from the coil‐compressed *k*‐space S(k)

  Estimate the rotation parameters ${\hat{\Theta }}_{R}$

  Apply ${\hat{\Theta }}_{R}$ to the *k*‐space $S_{m}\left(k\right)$

  Estimate the translation parameters ${\hat{\Theta }}_{T}$

  Apply ${\hat{\Theta }}_{T}$ to the rotation‐corrected *k*‐space $S_{m}\left(R^{T}k\right)$

  Apply the transformation parameters Θ to U(k).

**end for**

**Outputs**

$U_{c}\left(k\right)$: motion‐corrected multi‐coil undersampled *k*‐space

$\Theta =\left[\begin{array}{c} \Theta_{R}\\ \Theta_{T} \end{array}\right]$: rigid transformation parameters

Motion estimation and correction for each frame is performed in three steps: (1) the rotation is estimated and corrected in the coil‐compressed undersampled *k*‐space, (2) the translation is estimated and corrected in the rotation‐corrected coil‐compressed undersampled *k*‐space, and (3) a rigid transformation (i.e., rotation and translation) is applied to the multi‐coil undersampled *k*‐space. To avoid interpolation errors in *k*‐space, which would lead to blurring in the image, rotation is implemented as 3‐shears following the method proposed by Unser et al. [39] (see more details in Appendix A). A pseudocode description of the algorithm is included in Algorithm 1. The optimization was solved with a non‐linear conjugate gradient algorithm with backtracking line search [40].

### 3.3 Experiments

Our approach was compared with a robust groupwise image‐based registration method, pTVreg toolbox [18], whose input arguments were set up to perform rigid registration. The registration with pTVreg was performed from the SoS reconstructed dynamic images for the comparison with K‐CC‐MoCo. Also, rigid registration with pTVreg was performed from the fully‐sampled dynamic images for obtaining the best achievable MoCo (which can be considered a silver‐standard but, for simplicity, will be hereafter referred to as a reference MoCo). It is important to note that in these acquisitions the intra‐frame motion can be considered negligible even for fully‐sampled *k*‐spaces. Thus, the motion that we are correcting is the one occurring inter‐frames. In both cases, pTVreg was executed only on the previously defined ROI (Ω) to restrict the MoCo to the heart region in which the respiratory motion is highly rigid.

In addition, different evaluation metrics were used to compare the performance of K‐CC‐MoCo with respect to pTVreg on the undersampled DICOM dataset. Specifically, we computed the structural similarity index (SSIM) [41], the mean squared error (MSE), the high‐frequency signal‐to‐error ratio (HF‐SER) [42], and the perceptual sharpness index (PSI) [43]. The metrics were computed on the image obtained by adding the dynamic images across time in the aforementioned heart ROI (i.e., Ω). Thus, these metrics were computed between the motion‐corrected images (both with K‐CC‐MoCo and pTVreg) and the reference MoCo images (except for PSI, which is a non‐reference metric). For baseline comparison, the same metrics were also computed between the non‐motion‐corrected and the reference MoCo images. A Wilcoxon signed‐rank test was employed to evaluate the differences between the approaches.

The computational burden of both pTVreg and K‐CC‐MoCo was also evaluated. Mean execution times were measured across the 40 patients in the undersampled DICOM dataset. Both methods were executed on the same server (Intel(R) Xeon(R) CPU E5‐2697 v4@2.30 GHz, 482 GB RAM) under identical computational load conditions.

Furthermore, we segmented the myocardium of a few representative patients of the undersampled DICOM dataset. The myocardium is manually delineated on the last frame of the fully‐sampled dynamic image. Next, the rigid transformation estimated for the last frame is applied to the myocardium mask. For each pixel in the myorcardium, we obtain its time‐intensity curve; then, these curves are averaged across all the pixels in the same AHA segment [44] of the myocardium. For each averaged curve, say x[n],1≤n≤N, we have calculated its discrete cosine transform (DCT), X[k],1≤k≤N; these coefficients measure the contribution of a base of cosine functions with increasing frequency with the index k. Hence, the smoother the time‐intensity curve, the more energy will be concentrated in coefficients with lower k. Consequently, as a measure of curve smoothness we have represented the normalized cumulative energy of these coefficients, that is,

(9) $$E\left(j\right)=\frac{\sum_{k=1}^{j}X{\left[k\right]}^{2}}{\sum_{k=1}^{N}X{\left[k\right]}^{2}},1\leq j\leq N$$

where, by construction, 0≤E(j)≤1. It is expected that MoCo makes E(j) grow faster than for curves from no motion‐corrected images, since curves from the former are expected to have more low‐frequency content, while curves from the latter should be more ragged due to pixel incoherence (and hence, will have higher frequency content).

Finally, we investigate with the undersampled raw *k*‐space dataset whether elastic registration could improve the results compared to the proposed rigid correction in *k*‐space. To this end, we compare a pipeline consisting of a cascade of K‐CC‐MoCo (i.e., rigid MoCo in *k*‐space) followed by an L + S reconstruction [8], to a pipeline of an L + S reconstruction followed by a pairwise elastic registration in image space [21]. The elastic registration utilized was a multimodal pairwise approach based on NCC. Frame 40, after myocardium enhancement, was used as the reference.

## 4 Result

### 4.1 Digital Phantom

**FIGURE 2:**
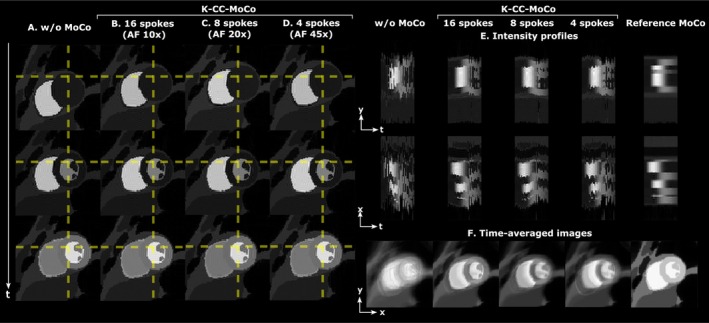
Digital phantom motion correction (MoCo) using the K‐CC‐MoCo approach for radial undersampling with acceleration factors (AFs) of approximately 10×, 20× and 45×. (A) Dynamic images without MoCo. (B) Dynamic images with K‐CC‐MoCo for 10× acceleration. (C) Dynamic images with K‐CC‐MoCo for 20× acceleration. (D) Dynamic images with K‐CC‐MoCo for 45× acceleration. (E) Intensity profiles in *y*‐*t* (foot‐head) and *x*‐*t* (right–left) directions. (F) Average across the frames of the dynamic images without MoCo, with K‐CC‐MoCo for 10×, 20×, and 45× accelerations, and for the reference MoCo. Motion correction is estimated from the undersampled *k*‐space in K‐CC‐MoCo and from fully‐sampled images in the reference MoCo, but the estimated corrections are always shown in the fully‐sampled images to facilitate MoCo visualization. Note that in A–D time increases as shown in the arrow on the left.

Figure 2 shows, for the digital phantom with radial undersampling, the dynamic images with and without MoCo when motion was estimated from the coil‐compressed undersampled *k*‐space with three different AF (i.e., 10×, 20×, and 45×). Note that motion is estimated from the coil‐compressed undersampled *k*‐space, although the results are shown in the fully‐sampled images to facilitate visualization. In addition, Figures S1 and S2 show the motion correction results for the digital phantom with a golden‐angle pseudo‐spiral sampling [36] and a Gaussian variable‐density random undersampling, respectively. Regarding the analysis with different noise levels, Figure S3 shows boxplots of the differences between each estimated and corresponding ground‐truth parameter for different CNRs and AFs in simulations with random combinations of rotations and translations in the digital phantom with radial undersampling. Figure S4 shows the estimated rotation ($\theta_{1}$) and translation ($\theta_{2}$ and $\theta_{3}$) parameters using K‐CC‐MoCo with respect to the ground‐truth (GT) parameters for different AFs in simulations representing shallow and deep breathing patterns.

### 4.2 Undersampled DICOM Dataset

**FIGURE 3:**
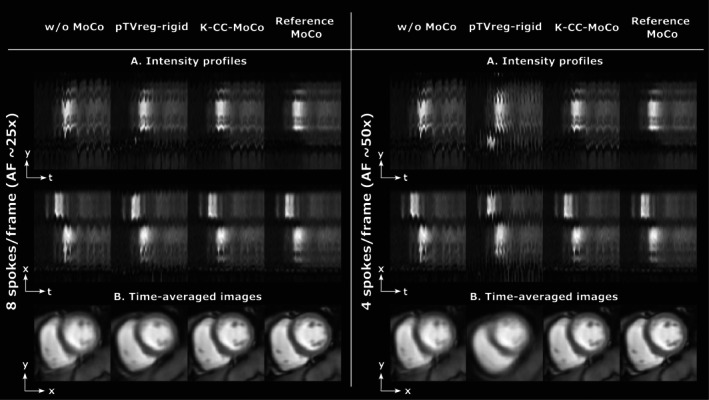
Motion correction (MoCo) of a REST acquisition for a representative patient for acceleration factors (AFs) of approximately 25× and 50×. (A) Intensity profiles in *y*‐*t* (foot‐head) and *x*‐*t* (right–left) directions. (B) Average across the frames of the dynamic images. Motion correction is estimated from the SoS image in pTVreg, from the undersampled *k*‐space in K‐CC‐MoCo, and from fully‐sampled images in the reference MoCo, but the estimated corrections are always shown in the fully‐sampled images to facilitate MoCo visualization. See Figure S5 for an extended version of this figure with the profile definitions.

**FIGURE 4:**
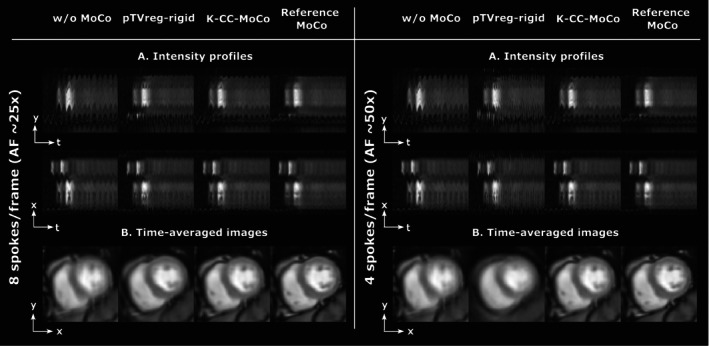
Motion correction (MoCo) of a STRESS acquisition for a representative patient for acceleration factors (AFs) of approximately 25× and 50×. (A) Intensity profiles in *y*‐*t* (foot‐head) and *x*‐*t* (right–left) directions. (B) Average across the frames of the dynamic images. Motion correction is estimated from the SoS image in pTVreg, from the undersampled *k*‐space in K‐CC‐MoCo, and from fully‐sampled images in the reference MoCo, but the estimated corrections are always shown in the fully‐sampled images to facilitate MoCo visualization. See Figure S6 for an extended version of this figure with the profile definitions.

Figure 3 shows the dynamic REST images with MoCo performed with K‐CC‐MoCo compared with pTVreg for a representative patient for different AFs (i.e., 25× and 50×). Similar information can be found in Figure 4, but for the dynamic STRESS images. Extended versions of these figures are provided in Figures S5 and S6, respectively.

**FIGURE 5:**
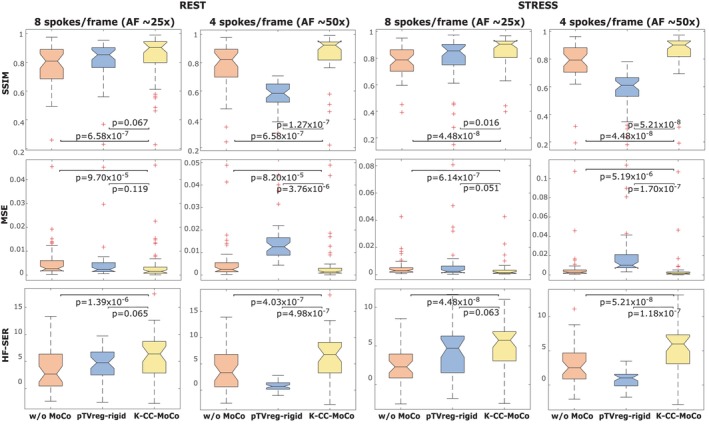
Boxplots for both REST and STRESS acquisitions and two different accelerations (approximately 25× and 50×) of structural similarity index (SSIM), mean squared error (MSE), and high‐frequency signal‐to‐error ratio (HFSER). The metrics were computed for the fully‐sampled images without MoCo, with pTVreg rigid MoCo, and with K‐CC‐MoCo. In all the cases, the metrics were calculated with respect to the fully‐sampled images with the reference MoCo. *p*‐values from Wilcoxon signed‐rank tests are reported.

Figure 5 shows boxplots of the evaluation metrics (i.e., SSIM, MSE, and HF‐SER) computed using all patients of this dataset, for the fully‐sampled images without MoCo, and the fully‐sampled images with K‐CC‐MoCo and pTVreg. In all the cases, the metrics were calculated with respect to the fully‐sampled images with the reference MoCo. Boxplots of the PSI evaluation metric are shown in Figure S7. Note that motion is estimated from the SoS image in pTVreg, and from the coil‐compressed undersampled *k*‐space in K‐CC‐MoCo, but results are shown in the fully‐sampled images to facilitate evaluation of the MoCo. Remarkably, although the performance of pTVreg is comparable to that of K‐CC‐MoCo at an AF of 25×, it decreases substantially as the AF increases to 50× for both REST and STRESS.

Regarding computational burden, the mean execution time for pTVreg in the REST acquisition was 62.95 and 53.33 s for AFs of 25× and 50×, respectively, while for K‐CC‐MoCo, the execution times were 32.00 and 32.38 s. In the STRESS acquisition, pTVreg required 82.80 and 70.68 s for AFs 25× and 50×, respectively, whereas K‐CC‐MoCo achieved execution times of 45.19 and 41.88 s. These timings correspond to the duration needed to estimate and correct all frames of a single slice. Notably, K‐CC‐MoCo is almost twice as fast as pTVreg.

**FIGURE 6:**
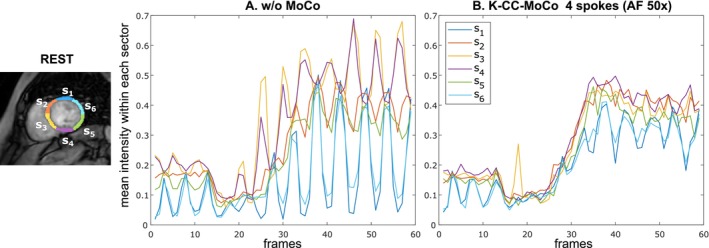
Perfusion intensity curves over time for six segments of the myocardium mask obtained for the REST acquisition from a representantive patient. The mean intensity values are computed in the segments of the myocardium mask for the fully‐sampled images (A) without MoCo and (B) with K‐CC‐MoCo. See Figure S8, which shows cumulative energy E(j) of each segment within the myocardium mask.

**FIGURE 7:**
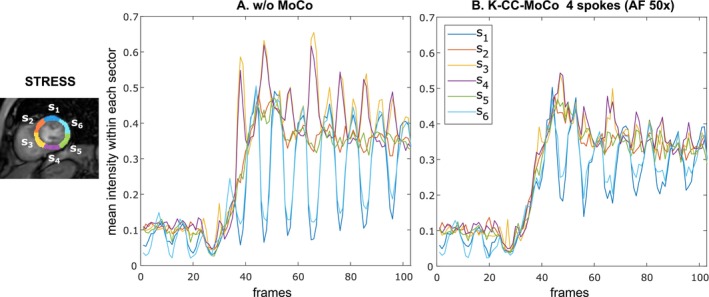
Perfusion intensity curves over time for six segments of the myocardium mask obtained for the STRESS acquisition from a representantive patient. The mean intensity values are computed in the segments of the myocardium mask for the fully‐sampled images (A) without MoCo and (B) with K‐CC‐MoCo. See Figure S9, which shows cumulative energy E(j) of each segment within the myocardium mask.

Figure 6 presents the time‐intensity curves for the segments of the myocardium mask of the fully‐sampled images, both with and without MoCo, for a REST acquisition from a representative patient. Additionally, Figure 7 displays similar information for the STRESS acquisition. Figures S8 and S9 show cumulative energy E(j) as a function of the coefficient index j (see Equation 9) for the segments on the myocardium masks s1–s6 defined in Figures 6 and 7, respectively.

### 4.3 Undersampled Raw *k*‐Space Dataset

**FIGURE 8:**
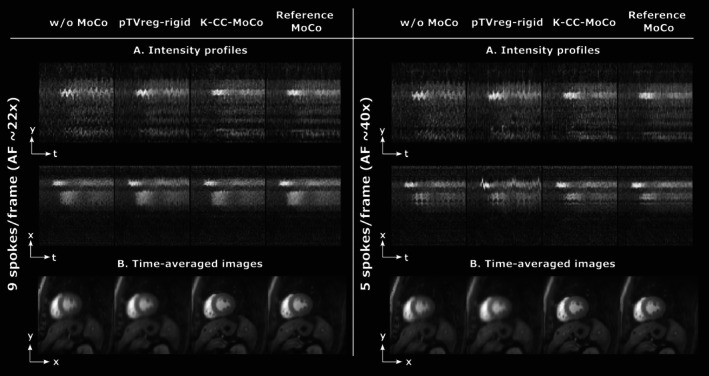
Motion correction (MoCo) in the mid slice of the raw *k*‐space dataset patient for two different accelarations, namely, nine spokes/frame and five spokes/frame (i.e., AF of 22× and 40×, respectively). (A) Intensity profiles in *y*‐*t* (foot‐head) and *x*‐*t* (right–left) directions. (B) Average across the frames of the dynamic images. Motion correction is estimated from the SoS image in pTVreg, from the undersampled *k*‐space in K‐CC‐MoCo, and from fully‐sampled images (i.e., 27 spokes per frame) in the reference MoCo, but the estimated corrections are always shown in the fully‐sampled images to facilitate visualization of the MoCo. See Figure S10 for an extended version of this figure with the profile definitions. Note that the effective temporal resolution for these accelerations correspond to approximately 23.04 and 12.8 ms for nine and five spokes per frame, respectively.

**FIGURE 9:**
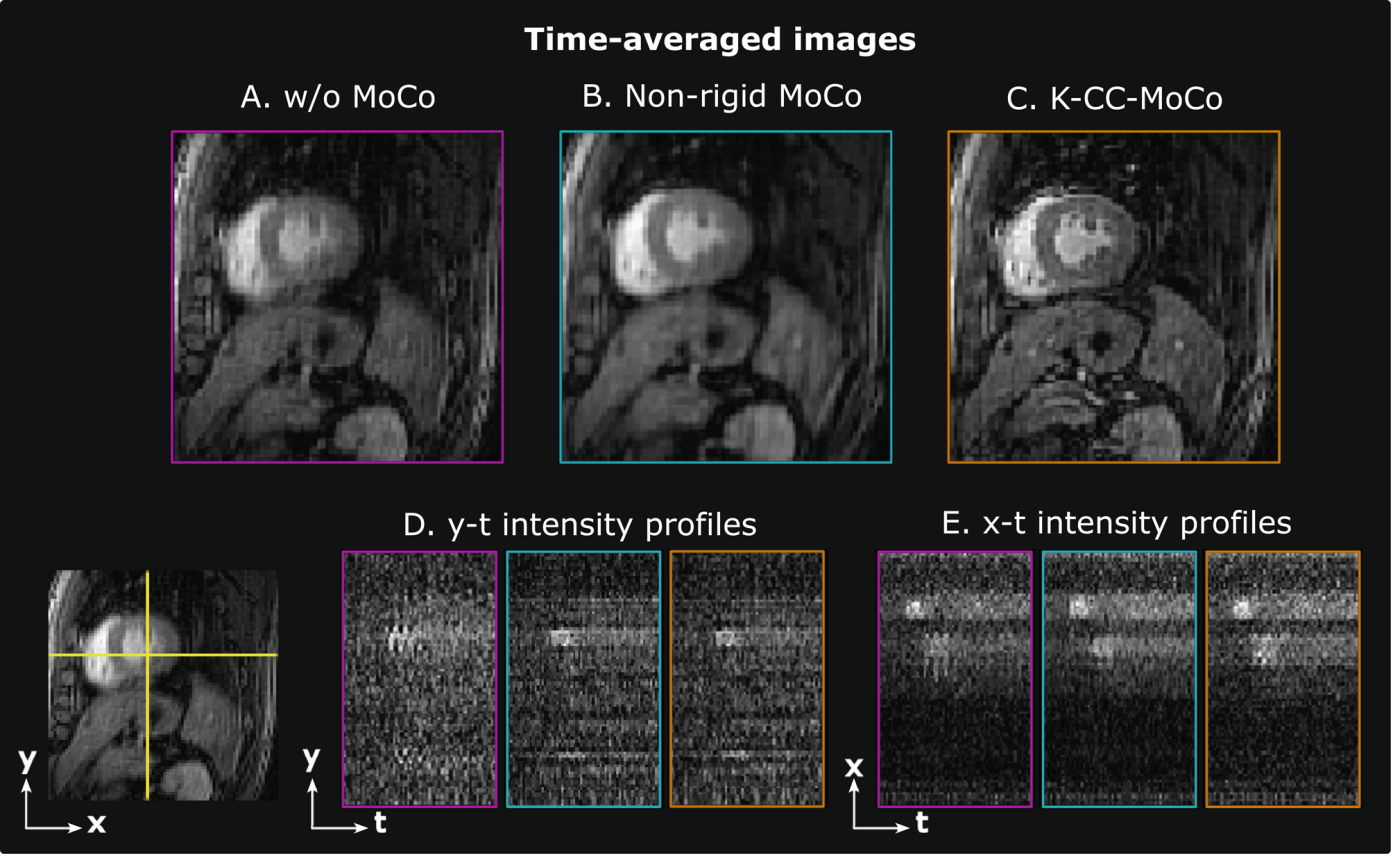
L + S reconstruction with different motion correction (MoCo) strategies in the raw *k*‐space dataset patient with acceleration of 14 spokes/frame (i.e., AF of 15×). Average across the frames for: (A) the reconstructed dynamic images without MoCo, (B) the dynamic images with the elastic MoCo in image space, and (C) the dynamic images with the proposed rigid MoCo in *k*‐space with K‐CC‐MoCo. (D) Intensity profiles in *y*‐*t* (foot‐head) direction. (E) Intensity profiles in *x*‐*t* (right–left) direction. Note that the effective temporal resolution for this acceleration corresponds to approximately 35.98 ms.

Figure 8 shows the mid slice of the dynamic REST images with MoCo performed with K‐CC‐MoCo compared with pTVreg. The results are shown for two different accelerations (i.e., 9 and 5 spokes per frame). An extended version of this figure is provided in Figure S10. Similar results but for the apical and basal slices are shown in Figures S11 and S12, respectively. Figure 9 shows for 14 spokes per frame a comparison between an L + S reconstruction pipeline with K‐CC‐MoCo (i.e., rigid MoCo in *k*‐space) and with elastic MoCo in image space. Finally, Figure S13 illustrates the cost function values for the L + S reconstruction method with and without the preceding K‐CC‐MoCo step.

## 5 Discussion

In this work, we have proposed K‐CC‐MoCo, an inter‐frame rigid MoCo approach for highly accelerated FPP‐CMR acquisitions formulated exclusively in *k*‐space. K‐CC‐MoCo was tested using a digital phantom and real patient data (both an undersampled DICOM dataset and, also, a raw *k*‐space dataset).

The digital phantom results demonstrate that the application of K‐CC‐MoCo notably reduces motion distortions. As expected, its performance tends to decline as the AF increases; nevertheless, the correction remains evident even in the presence of substantial motion and severe undersampling, with as few as four spokes per frame. This enhanced image quality would improve the diagnostic accuracy of various cardiovascular diseases characterized by perfusion abnormalities, including epicardial coronary artery disease, microvascular dysfunction, and certain cardiomyopathies [1]. The simulations with the asymmetric sawtooth pattern in the foot‐head translation parameter suggest that the approach remains relatively robust to different breathing patterns, including shallow, deep, and arrhythmic motion, although further validation with real data is required to confirm these observations.

Regarding the results with the undersampled DICOM dataset, the intensity profiles of Figures 3 and 4, corresponding to the REST and STRESS acquisitions, respectively, show how the application of the MoCo methods (both pTVreg and K‐CC‐MoCo) reduces variability across frames compared to the dynamic image without MoCo. Consequently, the resulting average images are notably less blurred. Comparing pTVreg and K‐CC‐MoCo, it is evident that as the acceleration factor increases, the correction performed by pTVreg deteriorates. At an AF of 50×, the resulting average image obtained with pTVreg is significantly distorted due to the presence of unusual transformations. This finding is also supported by the boxplots of the evaluation metrics shown in Figure 5. At 25× acceleration, the application of a MoCo approach (whether image‐based or *k*‐space‐based) improves the metrics compared to those obtained without MoCo. Specifically, no significant differences were found between pTVreg and K‐CC‐MoCo using the Wilcoxon test at this AF. However, at 50× acceleration, significant differences were observed between pTVreg and K‐CC‐MoCo on all metrics. Another important observation in Figures 3 and 4 is that time‐averaged images for the reference MoCo appear sharper because the frames are overall better aligned, but the intensity profiles show some degree of blurring in comparison with K‐CC‐MoCo probably as a result of the interpolation step that is not needed in the proposed method. The examples shown in Figures 6 and 7 also highlight a smoother behavior in the time‐intensity curves obtained from segments of the myocardium and their associated cumulative energy.

We have shown that the proposed *k*‐space‐based method, K‐CC‐MoCo, is approximately 2× faster than the image‐based method pTVreg, and can correct respiratory motion even at high AF, whereas the image‐based method fails in those cases due to strong undersampling artifacts. Although pTVreg with SoS reconstruction may misuse coil information, other reconstruction methods, such as CS and L + S, also suffer from artifacts and temporal blurring at high AFs and, they also require additional reconstruction time.

Results obtained for the raw *k*‐space data (Figure 8) show a similar behavior to that observed in both the phantom and the DICOM dataset. Similarly, the results obtained for the apical and basal slices (Figures S11 and S12) show corrections comparable to those observed in the mid slice. In addition, Figure 9 depicts that non‐rigid registration shows more blurring than K‐CC‐MoCo. Although the former is expected to be more flexible than the latter, a high AF could lead to poorer parameter estimation in the more complex model, resulting in a degradation of performance. Also, the interpolation step needed in the image‐based methods may play a role in the blurring visible in the time‐averaged images. Interestingly, performing motion correction prior to the reconstruction appears to improve the reconstruction itself by promoting sparsity in the temporal dimension [45]. This effect is depicted in the behavior of the L + S cost as a function of the iteration number, shown in Figure S13.

K‐CC‐MoCo has promising applications especially in deep learning and model‐based reconstructions, since motion could be directly estimated and corrected in *k*‐space without the need for an initial reconstruction, except for detecting the heart ROI which can be carried out using a rapid, low‐quality reconstruction. Thus, quantitative parameters could be directly estimated from *k*‐space [11], which may enable higher AFs. Also, physics‐informed deep learning models are typically trained with motion‐free images [10], which hinders their use in practice. This motion correction step in *k*‐space allows for a fast and straightforward implementation, and it could be easily added with minor changes as a pre‐processing step for all existing reconstruction workflows, with a low computational burden.

Motion estimation can also be performed using deep learning methods, as demonstrated in recent works [46], potentially accelerating the estimation process. Some approaches estimate motion directly in *k*‐space [31, 47] or jointly combine motion compensation and image reconstruction [48, 49]; however, limited research has focused on contrast‐enhanced acquisitions [50], particularly in first‐pass perfusion cardiac imaging.

This work has several limitations. Although K‐CC‐MoCo substantially reduces motion artifacts under high undersampling conditions, it does not completely eliminate them and it is outperformed by the image‐based registration method applied to fully‐sampled images. In our implementation, the heart ROI was empirically set to 80 × 80 pixels. Using an ROI size defined in physical units (e.g., in millimeters) would yield more consistent behavior across datasets and will be explored in future work. Nevertheless, the results across datasets with different in‐plane resolutions indicate that the proposed method is relatively robust to moderate variations in ROI size. Regarding the sampling requirements, the method can currently be applied only to Cartesian acquisitions due to the 3‐shears implementation of the rotation step; extending it to non‐Cartesian trajectories without re‐gridding would require additional modifications. Concerning the minimum information needed per frame for reliable motion estimation, the method is applicable to both time‐invariant and time‐varying sampling patterns; however, in the latter case, the limiting factor is the construction of the synthetic reference. For the specific case of radial sampling in a Cartesian grid, the effective AF was measured as the fraction of acquired samples within the *effective region*. This region was defined as the area around the center of *k*‐space where the Gaussian filter $G_{1}\left(k\right)$ reached 10% of its maximum value. The effective AF turned out to be below 2×.

Furthermore, the accuracy of the registration is limited by its sensitivity to through‐plane motion. While non‐rigid registration techniques offer a degree of flexibility by allowing local deformations, thereby enabling the alignment of, for example, heartbeats affected by mistriggering, rigid registration methods operate under the assumption of no deformation. As a result, rigid techniques are unable to accommodate anatomical changes across frames, which can lead to residual motion artifacts. This constraint reduces the effectiveness of rigid registration in scenarios where the heart exhibits significant morphological variation due to cardiac phase differences or arrhythmic events. Conversely, rigid motion correction methods, such as the one proposed, are generally more robust as can be seen in Figure 9. Also, rigid MoCo methods like K‐CC‐MoCo may serve as an initial step before applying non‐rigid correction, helping to prevent geometric distortions, even at low AF.

Future work includes additional tests using prospectively undersampled data with different acceleration rates to establish clinical applicability. The method should also be more exhaustively tested with other sampling strategies besides the radial (*k*,*t*)‐sampling scheme employed in this work. In this work, we have selected the NCC due to the fact that its definition in the image domain carries over directly to *k*‐space. However, the inclusion of a Gaussian term in Equation (6) departs from a direct subtraction of the mean in the image domain. A more general view leads to the question of whether a direct definition in *k*‐space of metrics customarily used in the image domain may prove competitive; this is also worth exploring for future work. Another potential extension of our method would be to incorporate a quality control or frame rejection mechanism; since the NCC value between each registered frame and the synthetic reference is available, this measure may serve as a diagnostic indicator of registration quality.

Finally, this work focused on rigid motion, because perfusion is usually measured approximately in the same cardiac phase across the contrast passage dimension, and 2D acquisition since clinical protocols generally comprise just a few short‐axis slices along the long axis. However, our mathematical formulation carries over to motion in 3D, and in the case that some jitter in the selected cardiac phase was observed, our model could be extended to affine motion due to the properties of the Fourier transform for variable transformation with a non‐unitary matrix. This extension also deserves further attention.

## 6 Conclusion

We propose K‐CC‐MoCo, an inter‐frame rigid respiratory MoCo for free‐breathing FPP‐CMR formulated in *k*‐space. The results obtained in a digital phantom and real patient data show that K‐CC‐MoCo outperforms image‐based correction for highly accelerated acquisitions, as high as 50×. Motion estimation directly from *k*‐space does not require high‐quality reconstructed dynamic images, unlike traditional methods that depend on such reconstructions.

## Funding

This work was supported by the LARSyS FCT (DOI: 10.54499/LA/P/0083/2020, 10.54499/UIDP/50009/2020, and 10.54499/UIDB/50009/2020), the Fundação para a Ciência e a Tecnologia (LA/P/0101/2020, UIDB/04326/2020, UIDP/04326/2020), the MICIU/AEI and the FSE+ (RYC2023‐045078‐I), the Ministerio de Ciencia e Innovación of Spain (PID2020‐115339RB‐I00, PID2022‐142166NA‐I00, TED2021‐130090B‐I00), and the ‘la Caixa’ Foundation (LCF/PR/HR22/00533, LCF/PR/HR22/52320018).

## Conflicts of Interest

J.S.G. is an employee of Philips.

## Supporting information


**Data S1:** Supporting Information.

## Data Availability

Research data are not shared.

## ENDNOTES

^*^
Noise standard deviation is computed based on the desired CNR and the contrast, which is determined by the maximum difference in myocardial signal along time during acquisition.

^†^
AF was calculated as the total number of points in the Cartesian grid divided by the non‐zero points.

## Appendix A Formulation

### Motion Correction Model

The corrected *k*‐space $S_{c}\left(k;\Theta \right)$ defined as in Equation (7), corresponds to the rigidly corrected version of the moving *k*‐space $S_{m}\left(k\right)$ where $\Theta_{R}=\left[\begin{array}{c} \theta_{1} \end{array}\right]$ and $\Theta_{T}={\left[\begin{array}{cc} \theta_{2} & \theta_{3} \end{array}\right]}^{T}$ are the parameters that define the rigid transformation for the particular case of a 2D correction. Thus, $R^{T}$ is the inverse of a rotation matrix with parameters $\Theta_{R}$. However, to avoid interpolation errors in *k*‐space, which lead to blurring in the image, rotation is implemented as 3‐shears following the method proposed by Unser et al. [39]

Thus, the rotation correction of the moving *k*‐space $S_{m}\left(k\right)$ for the particular case of 2D slices is implemented as

(A1)
$$S_{m}\left(R^{T}k\right)={ℱ}_{1}P_{2}{ℱ}_{1}^{H}{ℱ}_{2}P_{1}{ℱ}_{2}^{H}{ℱ}_{1}P_{2}{ℱ}_{1}^{H}S_{m}\left(k\right),$$

where ${ℱ}_{1}$ and ${ℱ}_{2}$ represent the Fourier transforms along columns and rows, respectively. The superscript H indicates the adjoint operator, that is, proportional to the inverse Fourier transform. Lastly, $P_{1}$ and $P_{2}$ are diagonal matrices, the entries of which are given by

(A2)
$$p_{1}=e^{-j\sin\left(\theta_{1}\right)k_{2}\cdot r_{1}}$$

(A3)
$$p_{2}=e^{j\tan\left(\theta_{1}/2\right)k_{1}\cdot r_{2}}$$

### Optimization Metric

Let the registration metric be defined in *k*‐space as

(A4)
$$\begin{array}{ll} & \text{GNCC}\left(S_{f}\left(k\right),S_{c}\left(k;\Theta \right)\right)=\frac{N\left(\Theta \right)}{D\left(\Theta \right)}=\\ & \frac{\sum_{k}\left(S_{f}\left(k\right)-S_{f}\left(k\right)G\left(k\right)\right)\left(S_{c}^{*}\left(k;\Theta \right)-S_{c}^{*}\left(k;\Theta \right)G\left(k\right)\right)}{\sqrt{\underset{E}{\underbrace{\sum_{k}{\left|S_{f}\left(k\right)-S_{f}\left(k\right)G\left(k\right)\right|}^{2}}}\;\underset{F}{\underbrace{\sum_{k}{\left|S_{c}\left(k;\Theta \right)-S_{c}(k;\Theta )G\left(k\right)\right|}^{2}}}}}\\ & =V\left(\Theta \right) \end{array}$$

Optimization may be carried out, alternatively, in ${\left|V\left(\Theta \right)\right|}^{2}$ to ensure that solutions based on gradient‐descent algorithms are real. In this case

(A5)
$$\Psi \left(\Theta \right)={\left|V\left(\Theta \right)\right|}^{2}=V\left(\Theta \right)V^{*}\left(\Theta \right)$$

Thus, the optimization problem is defined as follows:

(A6)
$$\begin{array}{cc} \hat{\Theta } & =\arg\min_{\Theta }\;-\Psi \left(\Theta \right)\\ & =\arg\min_{\Theta }\;-{\left|V\left(\Theta \right)\right|}^{2} \end{array}$$

### Derivatives

(A7)
$$\frac{\partial \Psi \left(\Theta \right)}{\partial \theta_{p}}=2ℜ\left\{\frac{\partial V\left(\Theta \right)}{\partial \theta_{p}}V^{*}\left(\Theta \right)\right\},$$

where ℜ{⋅} represents the real part operator.

(A8)
$$\begin{array}{cc} {\left[\nabla_{\Theta }V\left(\Theta \right)\right]}_{p} & =\frac{\partial V\left(\Theta \right)}{\partial \theta_{p}}=\frac{N^{\prime }\left(\Theta \right)}{D\left(\Theta \right)}-\frac{N\left(\Theta \right)D^{\prime }\left(\Theta \right)}{D^{2}\left(\Theta \right)}\\ & =\frac{N^{\prime }\left(\Theta \right)}{D\left(\Theta \right)}-\frac{N\left(\Theta \right)}{D^{2}\left(\Theta \right)}\frac{EF^{\prime }\left(\Theta \right)}{2D\left(\Theta \right)}\\ & =\frac{N^{\prime }\left(\Theta \right)}{D\left(\Theta \right)}-\frac{N\left(\Theta \right)E}{2D^{3}\left(\Theta \right)}F^{\prime }\left(\Theta \right) \end{array}$$

Since the transformation we are considering is rigid, we will assume that F will not change upon a change in $\Theta_{T}$. Thus, the derivative of F with respect to any parameter $\theta_{1+i}$ with 1≤i≤2 will considered to be zero.

Using the definition of $S_{c}\left(k;\Theta \right)$ in Equation (7), we can express the three derivatives as:

(A9)
$$\begin{array}{c} \text{1)}\frac{\partial N\left(\Theta \right)}{\partial \theta_{1}}=\sum_{k}{\left|1-G\left(k\right)\right|}^{2}S_{f}\left(k\right)\frac{\partial S_{c}^{*}\left(k;\Theta \right)}{\partial \theta_{1}}\\ =\sum_{k}{\left|1-G\left(k\right)\right|}^{2}S_{f}\left(k\right)\left(\frac{\partial S_{m}^{*}\left(R^{T}k\right)}{\partial \theta_{1}}e^{-j2\pi \Theta_{T}^{T}k}\right) \end{array}$$

with

(A10)
$$\begin{array}{ll} \frac{\partial S_{m}^{*}\left(R^{T}k\right)}{\partial \theta_{1}} & ={ℱ}_{1}\left(\frac{\partial P_{2}}{\partial \theta_{1}}{ℱ}_{1}^{H}{ℱ}_{2}P_{1}{ℱ}_{2}^{H}{ℱ}_{1}P_{2}\right.+P_{2}{ℱ}_{1}^{H}{ℱ}_{2}\frac{\partial P_{1}}{\partial \theta_{1}}{ℱ}_{2}^{H}{ℱ}_{1}P_{2}\\ & \;\left.+P_{2}{ℱ}_{1}^{H}{ℱ}_{2}P_{1}{ℱ}_{2}^{H}{ℱ}_{1}\frac{\partial P_{2}}{\partial \theta_{1}}\right){ℱ}_{1}^{H}S_{m}^{*}\left(k\right), \end{array}$$

where the derivatives of the diagonal elements of $P_{1}$ and $P_{2}$ are given by

(A11)
$$\frac{\partial P_{1}}{\partial \theta_{1}}=e^{-j\sin\left(\theta_{1}\right)k_{2}\cdot r_{1}}\left(-j\cos\left(\theta_{1}\right)k_{2}\cdot r_{1}\right)$$

(A12)
$$\frac{\partial P_{2}}{\partial \theta_{1}}=e^{j\tan\left(\theta_{1}/2\right)k_{1}\cdot r_{2}}\left(j\frac{1+\tan^{2}\left(\theta_{1}/2\right)}{2}k_{1}\cdot r_{2}\right)$$

(A13)
$$\begin{array}{ll} \text{2)}\frac{\partial N\left(\Theta \right)}{\partial \theta_{1+i}} & =\sum_{k}{\left|1-G\left(k\right)\right|}^{2}S_{f}\left(k\right)\frac{\partial S_{c}^{*}\left(k;\Theta \right)}{\partial u_{i}}\\ & \;=\sum_{k}{\left|1-G\left(k\right)\right|}^{2}S_{f}\left(k\right)\underset{\frac{\partial S_{c}^{*}\left(k;\Theta \right)}{\partial u_{i}}=\frac{\partial S_{c}^{*}\left(k;\Theta \right)}{\partial \theta_{1+i}}}{\underbrace{S_{m}^{*}\left(R^{T}k\right)e^{-j2\pi \Theta_{T}^{T}k}\left(-j2\pi {\left[k\right]}_{i}\right)}},\\ & 1\leq i\leq 2 \end{array}$$

(A14)
$$\begin{array}{cc} \text{3)}\frac{\partial F\left(\Theta \right)}{\partial \theta_{1}} & =\frac{\partial }{\partial \theta_{1}}\sum_{k}{\left|1-G\left(k\right)\right|}^{2}S_{c}\left(k;\Theta \right)S_{c}^{*}\left(k;\Theta \right)\\ & =\sum_{k}2{\left|1-G\left(k\right)\right|}^{2}ℜ\left\{\frac{\partial S_{c}\left(k;\Theta \right)}{\partial \theta_{1}}S_{c}^{*}\left(k;\Theta \right)\right\}, \end{array}$$

with $\frac{\partial S_{c}\left(k;\Theta \right)}{\partial \theta_{1}}$ as defined (but for the complex conjugate) in Equation (A9).
