# Supplementary material for: K‐CC‐MoCo: A Fast k‐Space‐Based Respiratory Motion Correction for Highly Accelerated First‐Pass Perfusion Cardiovascular MR
Source: Magn Reson Med. 2026 Feb 9;95(6):3536–49. doi: 10.1002/mrm.70287 (PMC13049264; doi:10.1002/mrm.70287)
Supplement: Supplementary file 1 — Data S1: Supporting Information. [file MRM-95-3536-s001.pdf]

Supporting Information: "K-CC-MoCo: a fast k-space-based respiratory motion correction for highly accelerated first-pass perfusion cardiovascular MR"

Manuscript Number: **MRM-25-26164**

December 29, 2025

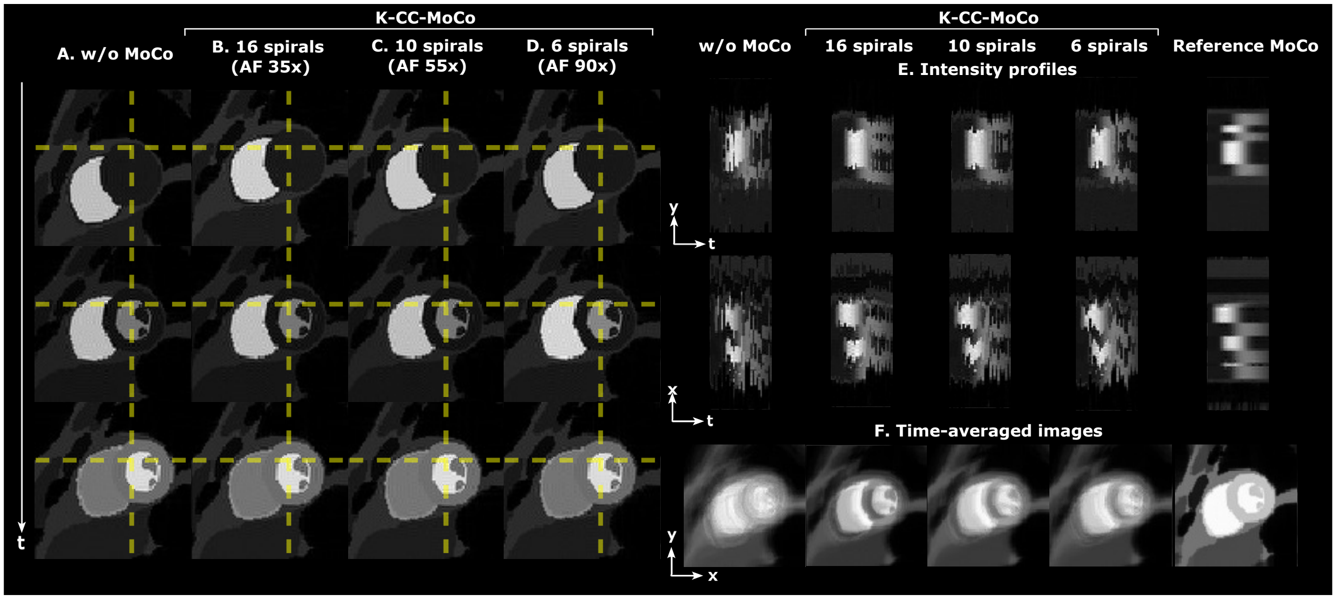

Figure S1: Digital phantom motion correction (MoCo) using the K-CC-MoCo approach for golden-angle pseudo-spiral sampling with acceleration factors (AFs) of approximately 35×, 55× and 90×. A) Dynamic images without MoCo. B) Dynamic images with K-CC-MoCo for 35× acceleration. C) Dynamic images with K-CC-MoCo for 55× acceleration. D) Dynamic images with K-CC-MoCo for 90× acceleration. E) Intensity profiles in y-t (foot-head) and x-t (right-left) directions. F) Average across the frames of the dynamic images without MoCo, with K-CC-MoCo for 35×, 55× and 90×. accelerations and with reference MoCo. Motion is estimated from the undersampled k-space, but results are shown in the fully-sampled images to facilitate MoCo visualization. Note that in A-D time increases as shown in the arrow on the left.

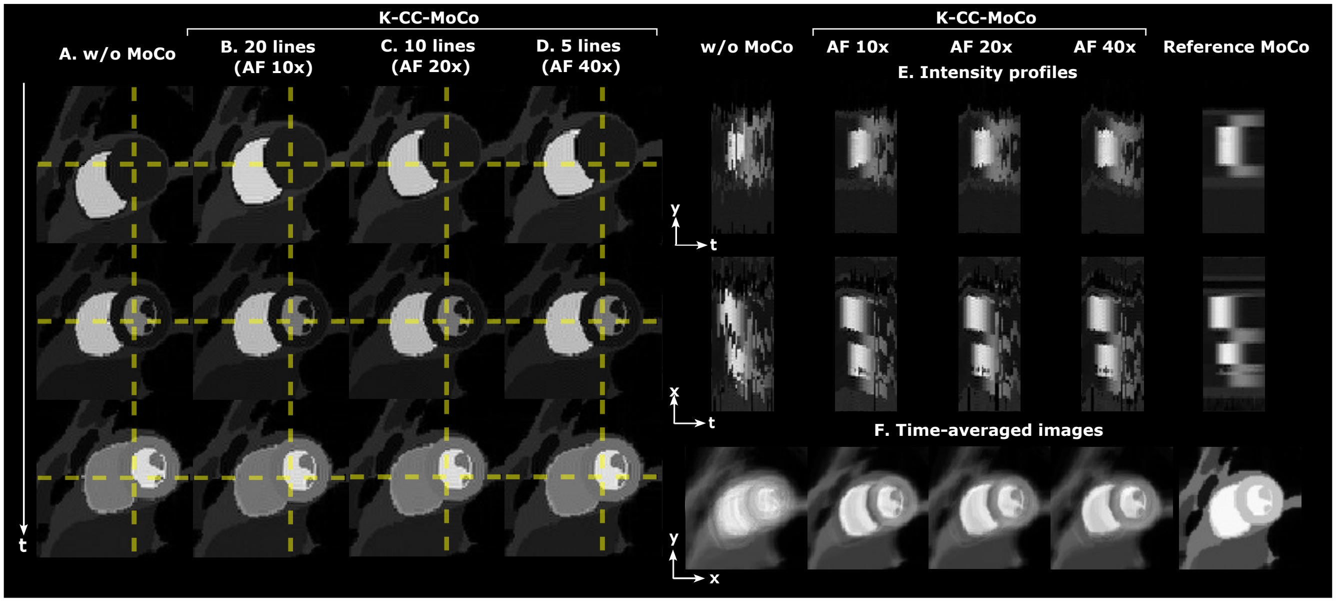

Figure S2: Digital phantom motion correction (MoCo) using the K-CC-MoCo approach for Gaussian variable-density random undersampling along the phase encoding direction with acceleration factors (AFs) of 10×, 20× and 40×. A) Dynamic images without MoCo. B) Dynamic images with K-CC-MoCo for 10× acceleration. C) Dynamic images with K-CC-MoCo for 20× acceleration. D) Dynamic images with K-CC-MoCo for 40× acceleration. E) Intensity profiles in y-t (foot-head) and x-t (right-left) directions. F) Average across the frames of the dynamic images without MoCo and with K-CC-MoCo for 10×, 20× and 40×. accelerations. Motion is estimated from the undersampled k-space, but results are shown in the fully-sampled images to facilitate MoCo visualization. Note that in A-D time increases as shown in the arrow on the left.

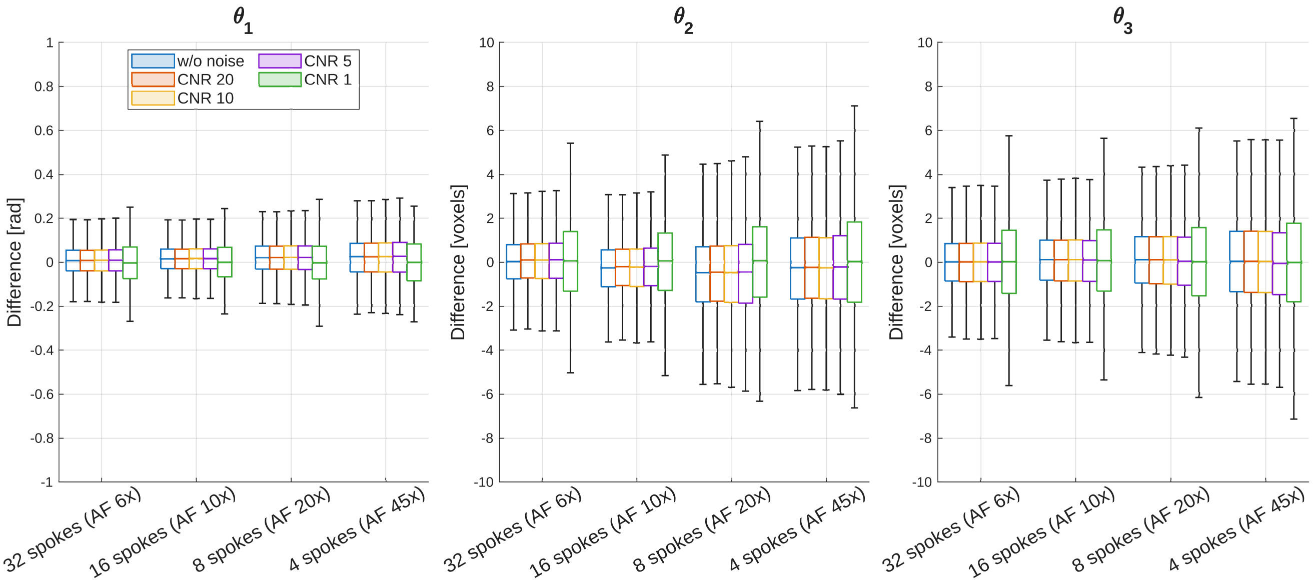

Figure S3: Boxplots of the differences between each estimated motion parameter and the corresponding ground-truth parameter in the digital phantom with random rotations and translations, for different contrast-to-noise (CNR) levels and acceleration factors (AFs) of radial sampling. Note that θ_1 corresponds to rotation, θ_2 to translation right-left, and θ_3 translation foot-head.

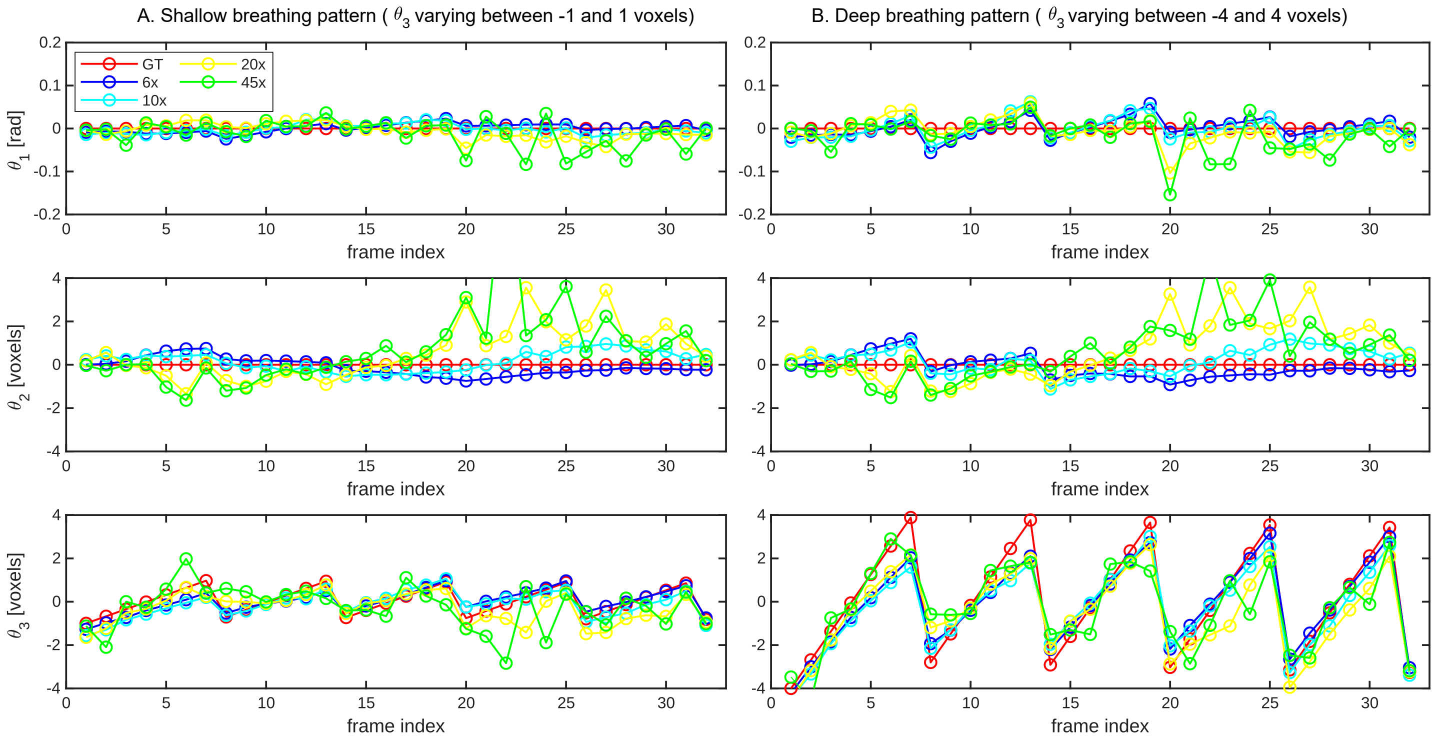

Figure S4: Temporal plots of the rotation (θ_1) and translation (θ_2 and θ_3) parameters estimated using K-CC-MoCo with respect to the ground-truth (GT) parameters for different acceleration factors (AFs) of radial sampling. To model different breathing patterns, asymmetric sawtooth variations were simulated in the foot–head translation parameter (θ_3), while the other two parameters were kept fixed at zero. Two different scenarios were simulated: A. θ_3 varied between -1 and 1 voxels to model shallow breathing (left column) and B. θ_3 varied between -4 and 4 voxels to model deep breathing (right column).

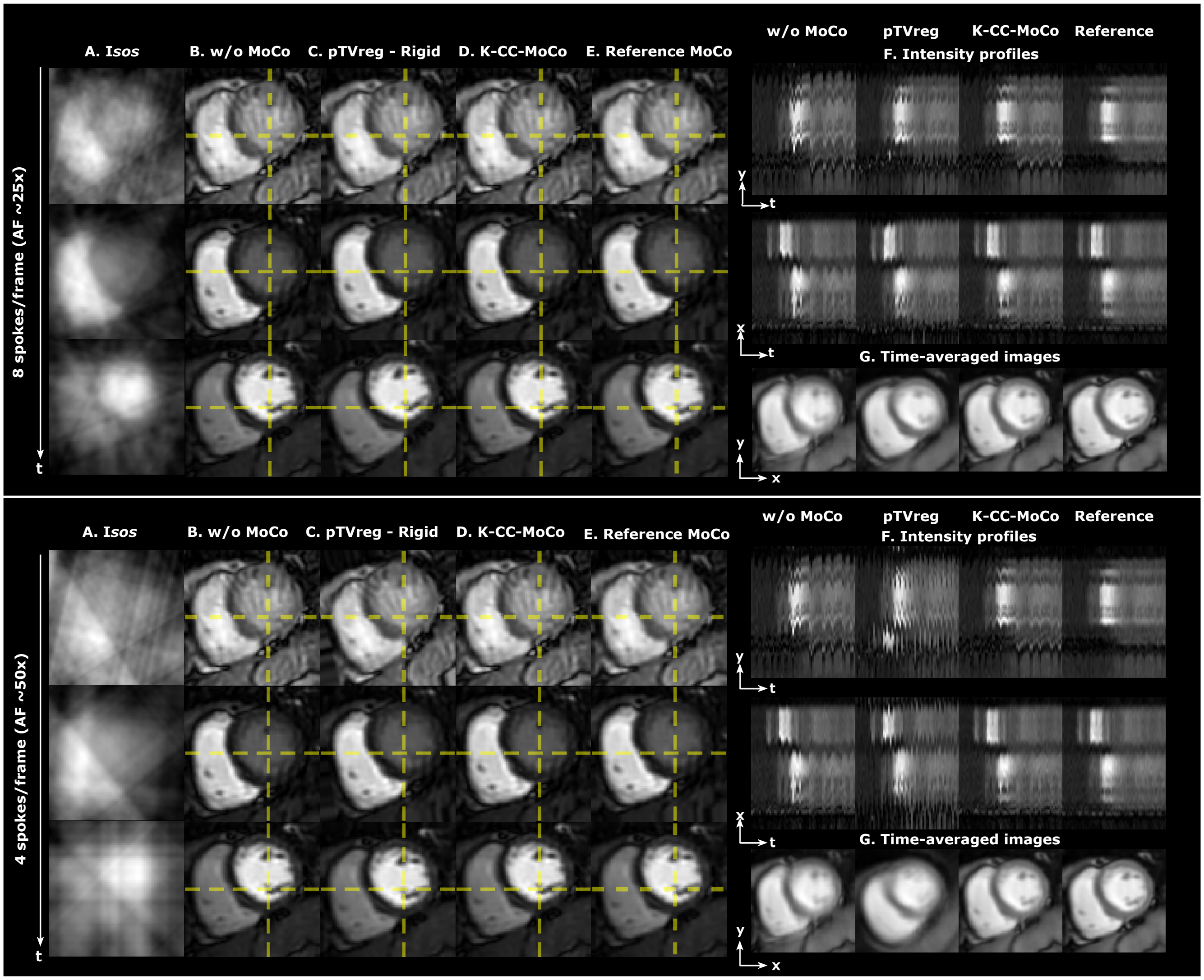

Figure S5: Motion correction (MoCo) of a REST acquisition for a representative patient for acceleration factors (AFs) of approximately 25× and 50×. A) Sum-of-squares (SoS) reconstructed image (I_{SoS}) from the multi-coil undersampled k-space. B) Dynamic images without MoCo. C) Dynamic images with image-based pTVreg rigid MoCo. D) Dynamic images with K-CC-MoCo. E) Dynamic images with reference MoCo. F) Intensity profiles in y-t (foot-head) and x-t (right-left) directions. G) Average across the frames of the dynamic images. Motion is estimated from the SoS image in C), and from the undersampled k-space in D), but results are shown in the fully-sampled images to facilitate MoCo visualization.

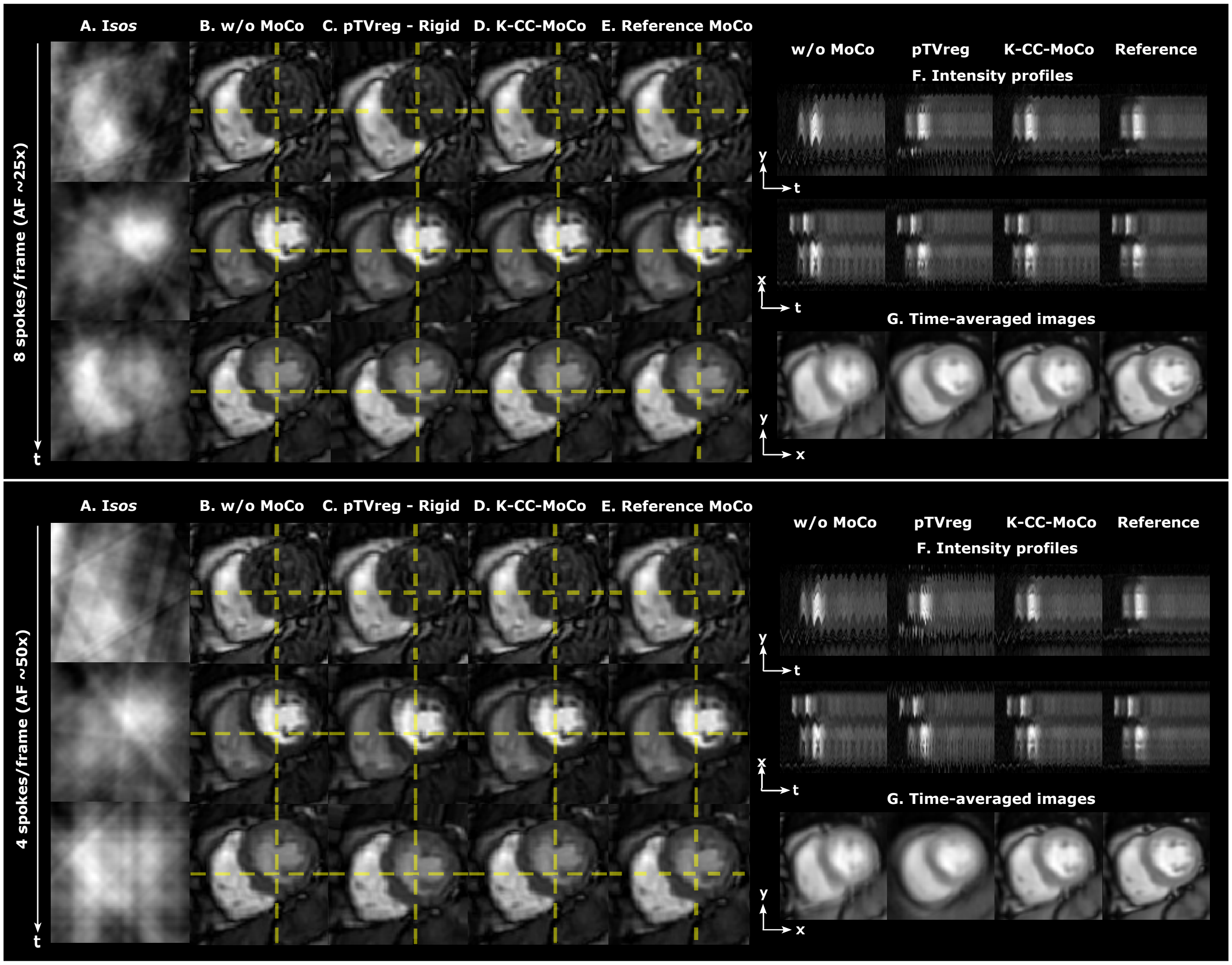

Figure S6: Motion correction (MoCo) of a STRESS acquisition for a representative patient for acceleration factors (AFs) of approximately 25× and 50×. A) Sum-of-squares (SoS) reconstructed image (I_{SoS}) from the multi-coil undersampled k-space. B) Dynamic images without MoCo. C) Dynamic images with image-based pTVreg rigid MoCo. D) Dynamic images with K-CC-MoCo. E) Dynamic images with reference MoCo. F) Intensity profiles in y-t (foot-head) and x-t (right-left) directions. G) Average across the frames of the dynamic images. Motion is estimated from the SoS image in C), and from the undersampled k-space in D), but results are shown in the fully-sampled images to facilitate MoCo visualization.

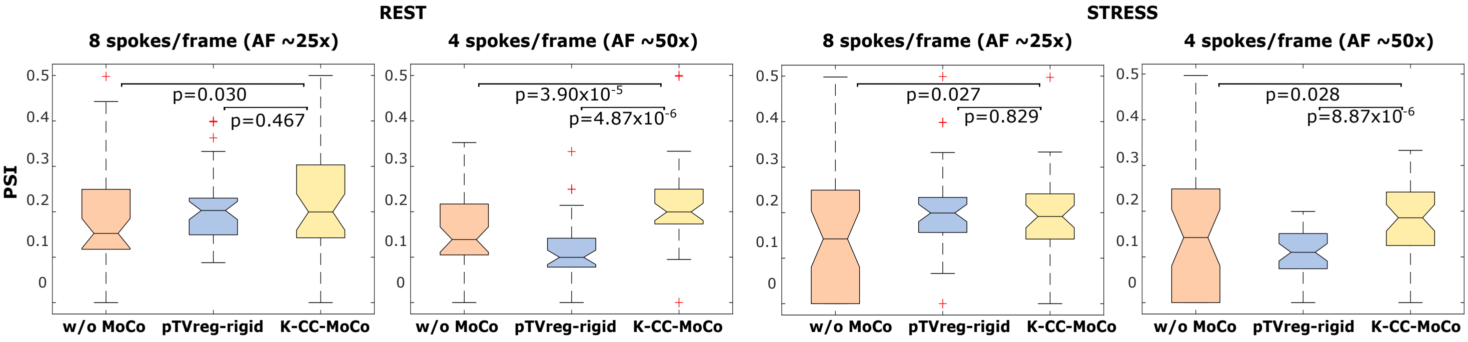

Figure S7: Boxplots for both REST and STRESS acquisitions and two different accelerations (approximately 25× and 50×) of the perceptual sharpness index (PSI) metric. The metrics were computed for the fully-sampled images without MoCo, with pTVreg rigid MoCo, and with K-CC-MoCo. p-values from Wilcoxon signed-rank tests are reported.

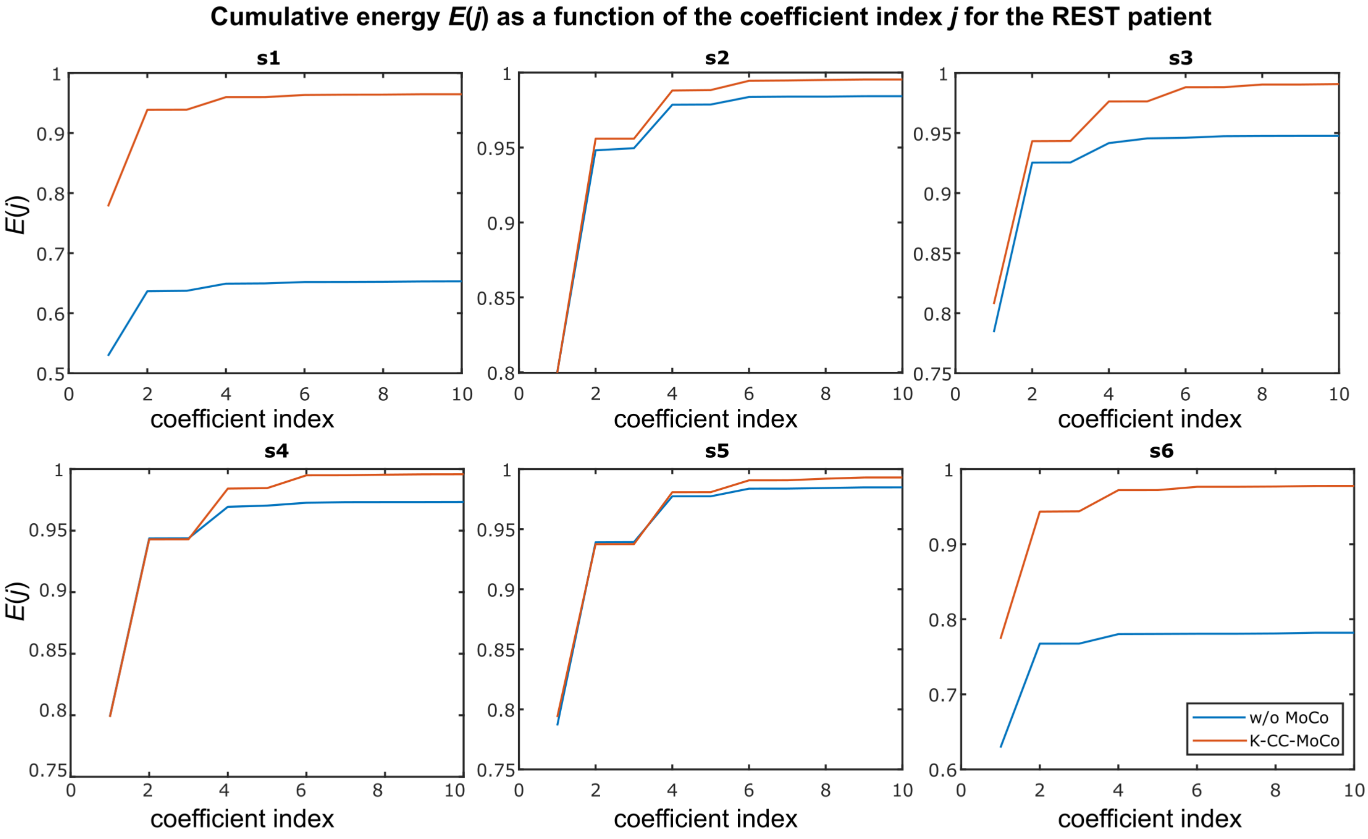

Figure S8: Cumulative energy $E(j)$ as a function of the coefficient index j (see Eq. 9) for the segments on the myocardium mask s1–s6 defined in the REST patient of Figure 6. With only 10 coefficients the cumulative energy is very close to unity for K-CC-MoCo curves while then non-corrected cases show lower values in four of the six segments, with s1 and s6 standing out.

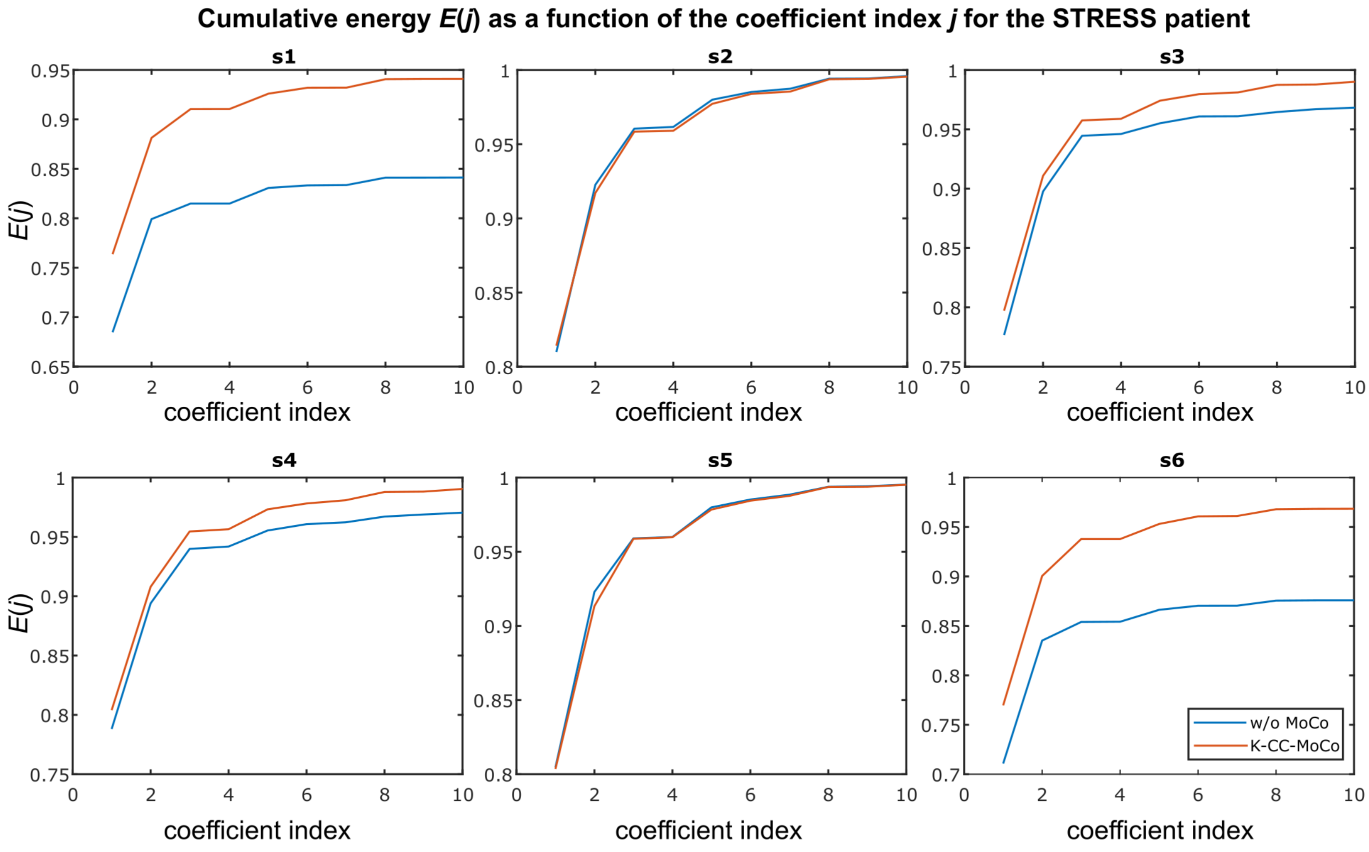

Figure S9: C) Cumulative energy $E(j)$ as a function of the coefficient index j (see Eq. 9) for the segments on the myocardium mask s1–s6 defined in the STRESS patient of Figure 7. Same comments as in Fig. S8 apply for the cumulative energy.

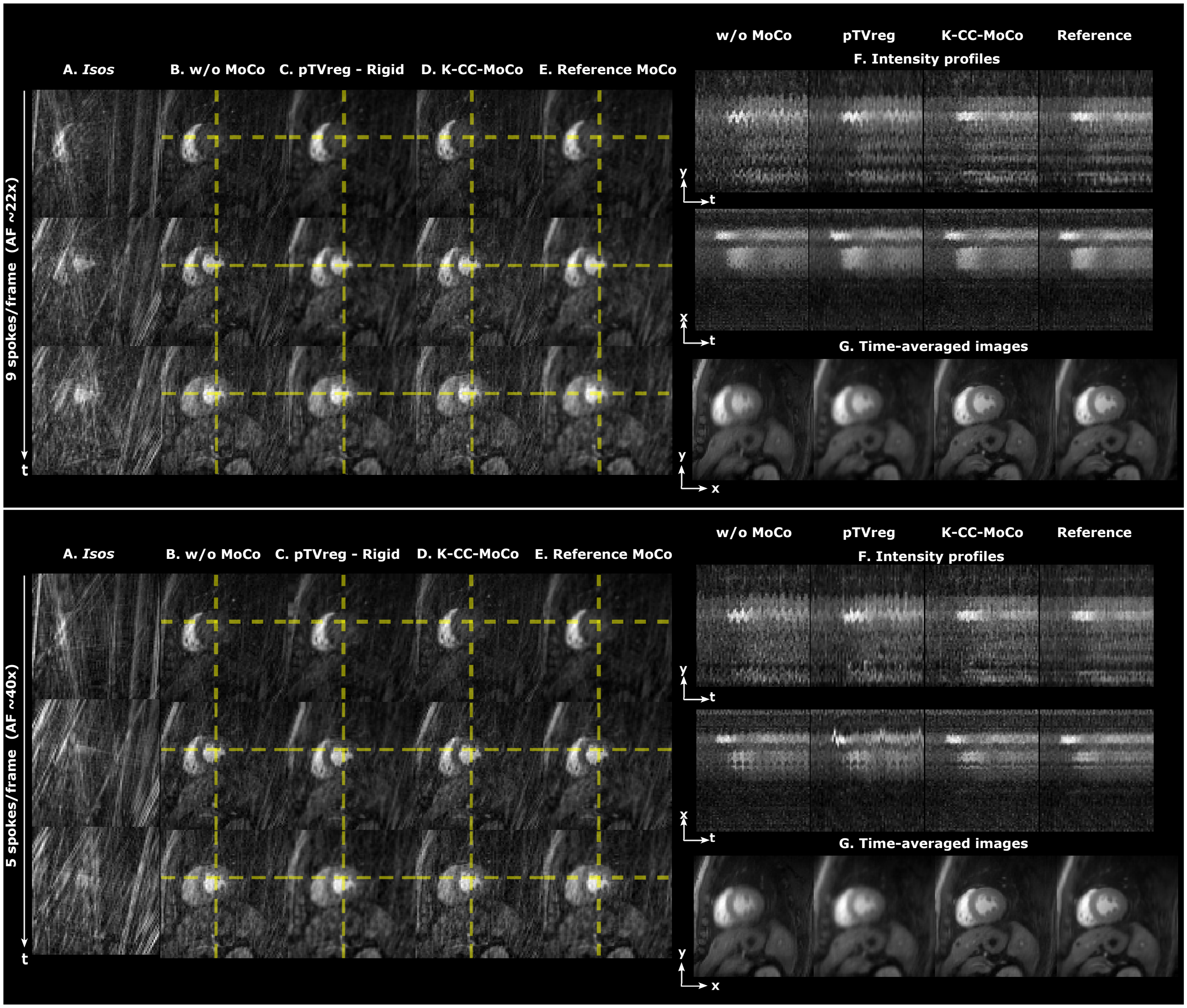

Figure S10: Motion correction (MoCo) in the mid slice of the raw k-space dataset patient for two different accelarations, namely, 9 spokes/frame and 5 spokes/frame. A) Sum-of-squares (SoS) reconstructed image (I_{SoS}) from the multi-coil undersampled k-space. B) Dynamic images without MoCo. C) Dynamic images with image-based pTVreg rigid MoCo. D) Dynamic images with K-CC-MoCo. E) Dynamic images with reference MoCo. F) Intensity profiles in y-t (foot-head) and x-t (right-left) directions. G) Average across the frames of the dynamic images. Motion is estimated from the SoS image in C), and from the undersampled k-space in D), but results are shown in the fully-sampled images (i.e., 27 spokes per frame) to facilitate visualization of the MoCo. Note that the effective temporal resolution for these accelerations correspond to approximately 23.04 ms and 12.8 ms for 9 and 5 spokes per frame, respectively.

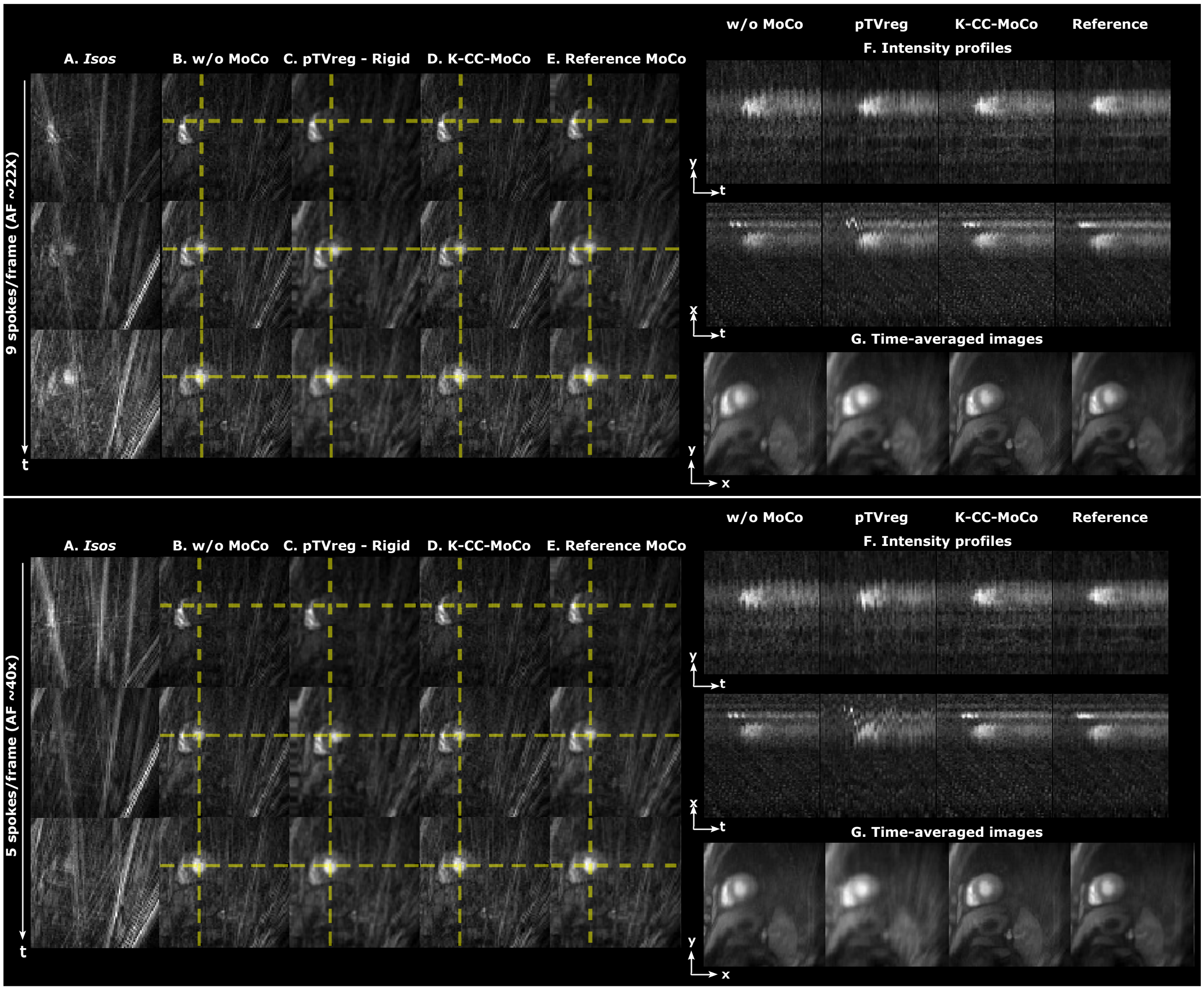

Figure S11: Motion correction (MoCo) in the apical slice of the raw k-space dataset patient for two different accelarations, namely, 9 spokes/frame and 5 spokes/frame. A) Sum-of-squares (SoS) reconstructed image (I_{SoS}) from the multi-coil undersampled k-space. B) Dynamic images without MoCo. C) Dynamic images with image-based pTVreg rigid MoCo. D) Dynamic images with K-CC-MoCo. E) Dynamic images with reference MoCo. F) Intensity profiles in y-t (foot-head) and x-t (right-left) directions. G) Average across the frames of the dynamic images. Motion is estimated from the SoS image in C), and from the undersampled k-space in D), but results are shown in the fully-sampled images (i.e., 27 spokes per frame) to facilitate visualization of the MoCo. Note that the effective temporal resolution for these accelerations correspond to approximately 23.04 ms and 12.8 ms for 9 and 5 spokes per frame, respectively.

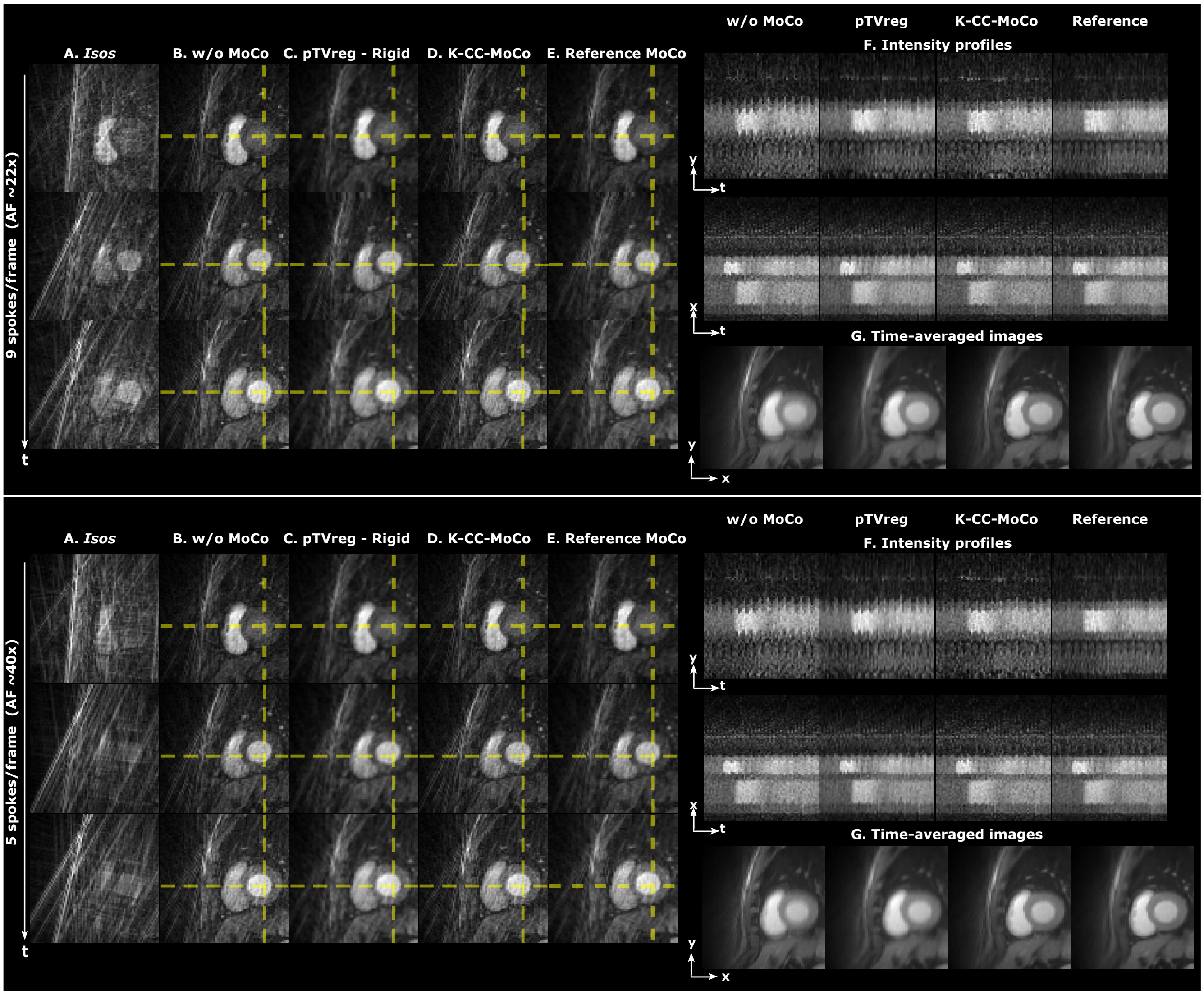

Figure S12: Motion correction (MoCo) in the basal slice of the raw k-space dataset patient for two different acce-larations, namely, 9 spokes/frame and 5 spokes/frame. A) Sum-of-squares (SoS) reconstructed image (I_{SoS}) from the multi-coil undersampled k-space. B) Dynamic images without MoCo. C) Dynamic images with image-based pTVreg rigid MoCo. D) Dynamic images with K-CC-MoCo. E) Dynamic images with reference MoCo. F) Intensity profiles in y-t (foot-head) and x-t (right-left) directions. G) Average across the frames of the dynamic images. Motion is estimated from the SoS image in C), and from the undersampled k-space in D), but results are shown in the fully-sampled images (i.e., 27 spokes per frame) to facilitate visualization of the MoCo. Note that the effective temporal resolution for these accelerations correspond to approximately 23.04 ms and 12.8 ms for 9 and 5 spokes per frame, respectively.

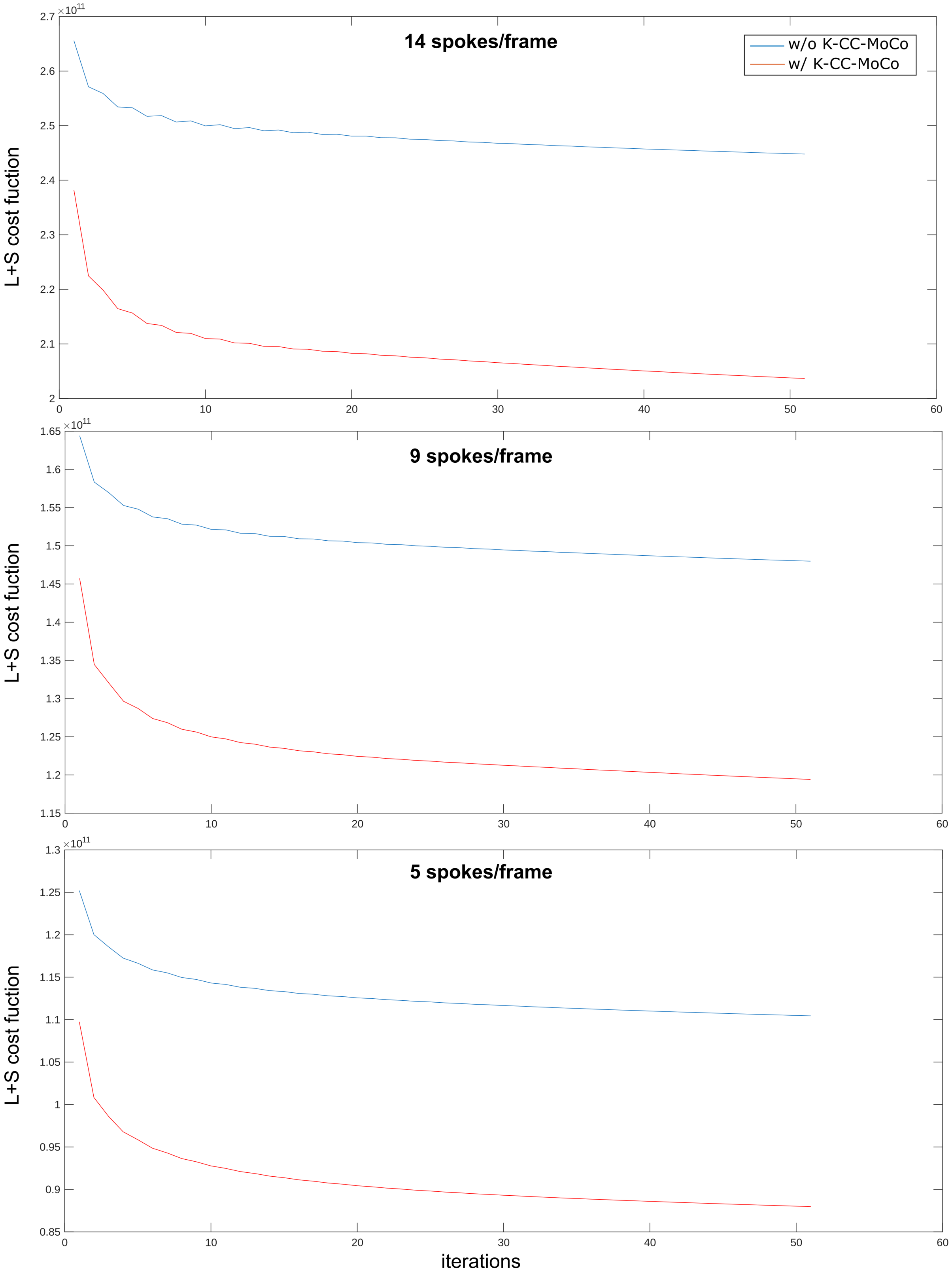

Figure S13: Cost function values of the L+S reconstruction method for the mid slice of the raw k-space dataset, shown for cases without and with the K-CC-MoCo step, for three accelerations (14, 9, and 5 spokes per frame).
